# Pseudo rotary resonance relaxation dispersion effects in isotropic samples

**DOI:** 10.5194/mr-6-119-2025

**Published:** 2025-06-03

**Authors:** Evgeny Nimerovsky, Jonas Mehrens, Loren B. Andreas

**Affiliations:** 1 Department of NMR-based Structural Biology, Max Planck Institute for Multidisciplinary Sciences, Am Faßberg 11, Göttingen, Germany

## Abstract

Enhanced transverse relaxation near rotary resonance conditions is a well-documented effect for anisotropic solid samples undergoing magic-angle spinning (MAS). We report transverse signal decay associated with rotary resonance conditions for rotating liquids, a surprising observation, since first-order anisotropic interactions are averaged at a much faster timescale compared with the spinning frequency. We report measurements of ${}^{13}C$ and ${}^{1}H$ signal intensities under spin lock for spinning samples of polybutadiene rubber, polyethylene glycol solution, and 99.96 % $D_{2}O$ . A drastic reduction in spin-lock signal intensities is observed when the spin-lock frequency matches 1 or 2 times the MAS rate. In addition, oscillations of the signal are observed, consistent with a coherent origin of the effect, a pseudo rotary resonance relaxation dispersion (pseudo-RRD). Through simulations, we qualitatively describe the appearance of pseudo-RRD, which can be explained by time dependence caused by sample rotation and an inhomogeneous field, the origin of which is an instrumental imperfection. Consideration of this effect is important for MAS experiments based on rotary resonance conditions and motivates the design of new MAS coils with improved radio frequency (RF)-field homogeneity.

## 1 Introduction

Measurement of the transverse relaxation rates of nuclear spins as a function of the applied RF-field spin-lock strengths is an elegant and well-established method for detecting structural molecular dynamics (Abyzov et al., 2022; Alam et al., 2024; Camacho-Zarco et al., 2022; Hu et al., 2021; Massi and Peng, 2018; Palmer, 2015; Palmer and Massi, 2006; Pratihar et al., 2016; Rangadurai et al., 2019; Sekhar and Kay, 2019; Stief et al., 2024). With magic-angle spinning (MAS) NMR, (Andrew et al., 1958; Lowe, 1959) rocking motion or slow exchange in molecular solids have been studied via the impact on transverse relaxation. (Fonseca et al., 2022; Keeler and McDermott, 2022; Krushelnitsky et al., 2018, 2023; Kurauskas et al., 2017; Lewandowski et al., 2011; Ma et al., 2014; Marion et al., 2019; Öster et al., 2019; Quinn and McDermott, 2009; Rovó and Linser, 2018; Shcherbakov et al., 2023; Vugmeyster et al., 2023). This detection can be achieved by performing a spin-lock experiment (Furman et al., 1998), where the decay of magnetization is measured as a function of the power of the applied spin-lock (SL) pulse. For slow motion or slow exchange in the microsecond ( µs ) range, the spectral densities (Redfield, 1957) of the investigated spins may include additional terms (Kurbanov et al., 2011; Marion et al., 2019) that arise from non-averaged anisotropic interactions (Kurbanov et al., 2011; Rovó, 2020; Schanda and Ernst, 2016). These terms depend on the sums and differences between the nutation frequency induced by the RF field ( $\nu_{\text{SL}}=\gamma B_{1}/(2\pi )$ ) and MAS rate ( $\nu_{R}$ ). Such dependence causes a significant increase in the measured relaxation rates when $\nu_{\text{SL}}$ approaches one of the rotary resonance conditions ( $\nu_{\text{SL}}=\nu_{R}$ or $2\nu_{R}$ ) (Marion et al., 2019).

For liquid samples, where SL experiments are routinely used to detect fast exchange (Cavanagh et al., 2006; Deverell et al., 1970; Palmer, 2004), sample rotation is not expected to induce any rotary resonance conditions based on anisotropic spin interactions (Levitt et al., 1988; Oas et al., 1988) since such interactions are eliminated by nanosecond-timescale isotropic motion (Haeberlen and Waugh, 1968; Maricq, 1982). However, to our surprise, we still observed changes in the SL signals at rotary resonance conditions for liquid and liquid-like samples during SL experiments. Since the signal decreases but is also clearly oscillatory, a signature of coherent effects, we refer to this phenomenon as a pseudo rotary resonance relaxation dispersion (pseudo-RRD). A review of the literature revealed articles suggesting related resonance conditions for rotating liquid samples: in adiabatic TOCSY (total correlation spectroscopy) experiments, enhanced performance was observed under specific matching conditions in relation to the spinning frequency (Kupče et al., 2001; Zektzer et al., 2005).

In this article, we measured pseudo-RRD for several liquid and liquid-like samples and observed similar effects in each. Through numerical simulations (Nimerovsky and Goldbourt, 2012), we show that this behavior can be qualitatively explained by the influence of the periodic component of the applied RF field, which arises from the rotation of the sample in a spatially inhomogeneous RF field (Aebischer et al., 2021; Tošner et al., 2017).

## 2 Results and discussion

We measured pseudo-RRD for natural-abundance ${}^{13}C$ polybutadiene rubber at 10, 20, and 35 kHz MAS. The same pseudo-RRD behavior is observed for a polyethylene glycol solution at 10 kHz MAS and for residual protons in liquid deuterium oxide (99.96 %). The polybutadiene rubber displays liquid-like spectra but does not undergo translational diffusion due to the elastomeric properties of a cross-linked polymer. On the other hand, since the polybutadiene is an elastomer and therefore may not undergo perfect isotropic averaging, we also recorded data for a polyethylene glycol solution and liquid water.

**Figure 1:**
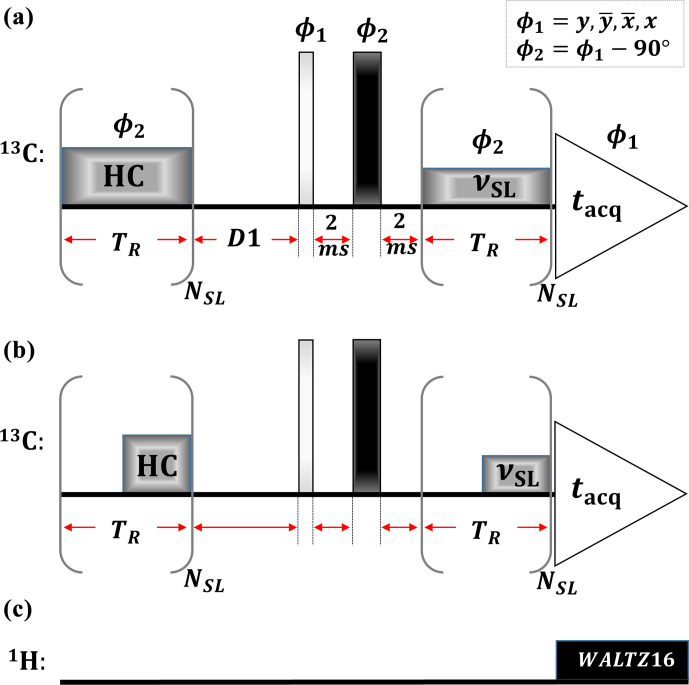
Spin-lock sequence with heat compensation (HC), $T_{2}$ filter (2 ms – π pulse – 2 ms), and spin-lock (SL) blocks. The SL and HC elements consisted of a train of $N_{\text{SL}}$ rotor-synchronized continuous **(a)** or windowed **(b)** pulses with the same phase ( $\phi_{2}$ ) and RF-field strength ( $\nu_{\text{SL}}$ ). In all experiments, ${\text{power}}_{\text{HC}}+{\text{power}}_{\text{SL}}=\text{constant}$ (equivalent to 50 kHz RF-field strength). During acquisition, WALTZ-16 decoupling (Shaka et al., 1983) **(c)** was applied to the ${}^{1}H$ channel.

**Figure 2:**
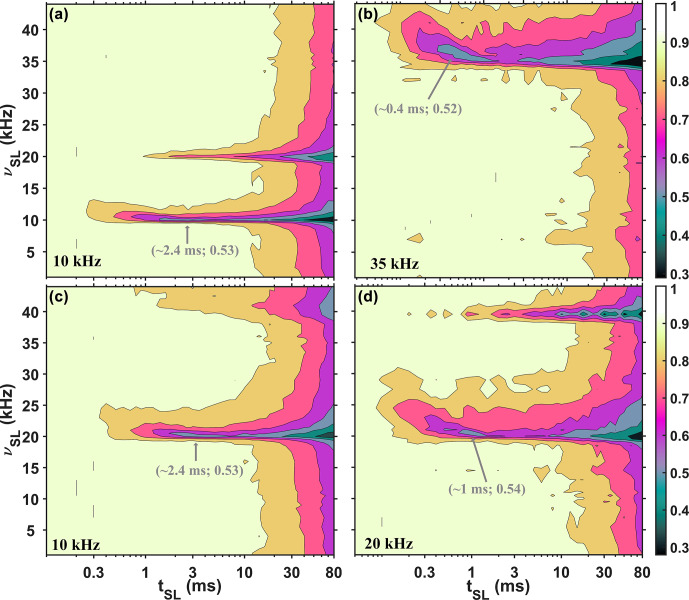
The ${}^{13}C$ polybutadiene rubber signal (the peak intensities) is shown as functions of the RF-field strength ( $\nu_{\text{SL}}$ , y axis) and mixing time ( $t_{\text{SL}}$ , x axis) of the SL under three different MAS rates: 10 kHz **(a, c)**, 20 kHz **(d)**, and 35 kHz **(b)**. For **(a)**, **(b)**, and **(d)**, continuous SL was applied, while for **(c)**, windowed (half of the rotor period was filled with the pulse) SL was implemented. The values in gray represent the coordinates of the first minimum in the profiles. Additional experimental details are provided in the Supplement.

Figure 1 displays the spin-lock sequence. Similar to previously proposed versions (Vugmeyster et al., 2022), it contains a heat compensation block (Wang and Bax, 1993) (HC), followed by a π/2 pulse, $T_{2}$ filter (Schmidt-Rohr et al., 1992) (to reduce any broad signal components from the polymer), and spin-lock pulse (SL). The mixing times for HC and SL pulses were the same during a single experiment ( $t_{\text{HC}}=t_{\text{SL}}=N_{\text{SL}}T_{R}$ ), while the sum of the RF-field powers of these applied pulses always equaled a fixed value. In all experiments, we used continuous HC and SL (Fig. 1b) except in one (the data are shown in Fig. 2c), where we applied windowed pulses (Fig. 1b). During acquisition, WALTZ16 decoupling (Shaka et al., 1983) was used.

The experimental ${}^{13}C$ polybutadiene rubber SL profiles (acquired with a 1.3 mm probe) under three different MAS rates of 10 kHz (a and c), 20 kHz (d), and 35 kHz (b) are shown in Fig. 2. For Fig. 2a, b, and d, a drastic reduction in the SL signal is observed at rotary resonance conditions when $\nu_{\text{SL}}$ equals either $\nu_{R}$ or $2\nu_{R}$ . Together with reduction in the SL signal, oscillations are observed. For Fig. 2c, we used 10 kHz MAS and windowed pulses: half of the rotor period is a window, as shown in Fig. 1b. Again, a drastic reduction in the SL signal is observed, but when $\nu_{\text{SL}}$ equals either $2\nu_{R}$ or $4\nu_{R}$ . We previously observed similar behavior for windowed cross-polarization (CP) profiles (Nimerovsky et al., 2023), where increasing the window between rotor-synchronized pulses from zero to half of a rotor period doubled the required RF-field strength for CP transfers (Hartmann and Hahn, 1962). Interestingly, with windowed pulses, the SL profile appears similar to that with continuous pulses, and even under a low RF-field strength of 1 kHz, there is no change in the SL signal intensities (Fig. S1a in the Supplement). The experimental spin-echo (Hahn, 1950) and inversion recovery (Vold et al., 1968) curves for this sample are illustrated in Fig. S1a and b.

From Fig. 2, we can also observe that the location of the first minimum signal intensity in the experimental SL profiles depends on the MAS rate (indicated in gray in Fig. 2). For 10 kHz MAS (Fig. 2a and c), the locations are at approximately a 3 ms SL time, while for 20 kHz (Fig. 2d) and 35 kHz (Fig. 2b), the locations are at approximately 1 and 0.4 ms, respectively. However, in all four profiles at these minimum points, the signal reaches a similar value of approximately 0.53.

**Figure 3:**
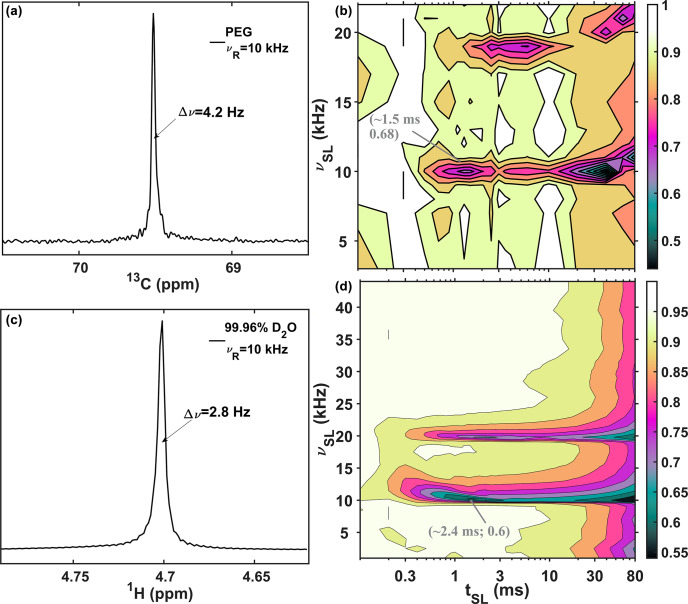
${}^{13}C$ and ${}^{1}H$ spin-lock profiles at 10 kHz MAS. **(a, b)** Single-pulse ${}^{13}C$ spectra and SL profile of polyethylene glycol (PEG) acquired with a 4 mm probe. **(c, d)** ${}^{1}H$ single pulse and SL profile of 99.96 % $D_{2}O$ acquired with a 1.3 mm probe. The profiles in **(b)** and **(d)** show ${}^{13}C$ and ${}^{1}H$ signal amplitudes (peak intensities) as a function of the RF-field strength ( $\nu_{\text{SL}}$ , y axis) and mixing time ( $t_{\text{SL}}$ , x axis) of the SL pulse. The values in gray are the coordinates of the first minimum in the profiles. Additional experimental details are provided in the Supplement.

Rotary resonance conditions at $\nu_{R}$ and $2\nu_{R}$ of RF-field strength are also observed for the polyethylene glycol (Fig. 3b, acquired with a 4 mm probe) and for residual protons in liquid deuterium oxide (Fig. 3d, acquired with a 1.3 mm probe). The 1D spectra of these samples are shown in Fig. 3a and c for PEG and liquid water. For each sample, two rotary resonance conditions are clearly observed at positions equal to integer multiplies of the MAS rates ( $\nu_{\text{SL}}=\nu_{R}$ , $2\nu_{R}$ ). For liquid water (Fig. 3d), the additional rotary resonance condition with n=3 appears very weak. We more carefully sampled around this condition for the water sample.

The performance of the SL experiments on all three samples helps rule out the influence of translational diffusion (Hahn, 1950) (which may be present for polyethylene glycol and liquid water but not for polybutadiene rubber) or residual dipolar interaction (Cohen-Addad and Vogin, 1974) (which might be present for polybutadiene rubber but is not relevant for polyethylene glycol and liquid water).

To identify the major source of the apparent rotary resonance conditions in liquid and liquid-like samples, we performed theoretical and numerical analysis of the spin-lock (SL) signal (Eqs. 1–7 below). In this analysis, three possible sources of pseudo-RRD are considered, all of which are time-dependent periodic functions. The first two are related to $B_{0}$ and $B_{1}$ modulations, which arise from the rotation of the sample within inhomogeneous $B_{0}$ or $B_{1}$ fields. Note that the $B_{0}$ field refers to the main field and that modulations in $B_{0}$ can be in any direction. Similarly, $B_{1}$ field modulations can occur in any direction, and z -direction modulations are certainly present for a solenoid at the magic angle. The precise distributions of $B_{0}$ or $B_{1}$ fields in MAS probes have been previously investigated (Engelke, 2002; Gupta et al., 2015; Hoult, 1976; Hürlimann and Griffin, 2000; Paulson et al., 2004; Tošner et al., 2017, 2018). Here we consider a simplified model of field distributions in order to reveal the qualitative dependence on MAS rates rather than predict the exact behavior of a particular probe. Note that the consideration of spatially distributed $B_{0}$ field inhomogeneity is compatible with a narrow line width under MAS (Sodickson and Cory, 1997). For completeness of the theoretical analysis, a dipolar interaction between a pair of spins was also included as a third possible source, although it may be disregarded since the rotary resonance effect was observed for ${}^{1}H$ spins in 99.96 % $D_{2}O$ (Fig. 3a and b).

The effects of an inhomogeneous RF field on MAS spectra have been investigated previously (Aebischer et al., 2021; Goldman and Tekely, 2001; Tekely and Goldman, 2001; Tošner et al., 2017). Rather than $B_{1}$ oscillations, the coil receptivity was shown to oscillate due to rotation of the sample relative to the coil, and the authors showed that this instrumental imperfection results in the appearance of sidebands that are unrelated to the chemical shift anisotropy (CSA) (Goldman and Tekely, 2001; Tekely and Goldman, 2001). Sidebands due to rotation through inhomogeneous $B_{0}$ and $B_{1}$ fields are a well-known effect in liquids (Malinowski and Pierpaoli, 1969; Vera and Grutzner, 1986). For solid samples, Aebischer et al. (2021) investigated the influence of time-dependent modulations of the RF-field amplitude and phase on the performance of selected recoupling sequences and nutation experiments. In this case, the modulations did not significantly affect most recoupling sequences, with the exception of double quantum C-symmetry sequences (Lee et al., 1995). It was noted much earlier that oscillations in phase were needed to fully explain experimental results in rotary resonance recoupling (Levitt et al., 1988). Consistent with the matching conditions identified in this study, Aebisher et al. (2021) revealed significant effects at $\nu_{R}$ and $2\nu_{R}$ in nutation spectra. The distribution of $B_{1}$ fields in a solenoidal coil was elegantly visualized in SL experiments of solid samples, in which case the loss of signal at rotary resonance was interpreted as CSA recoupling (Tošner et al., 2017).

To understand the origin of the pseudo-RRD effect, we start with the simplest case, investigating the behavior of an on-resonance spin ( I ) during the RF-field spin lock. The simulated SL signal is defined as follows:

1 $$S_{\text{SL}}(t_{\text{SL}})=\text{Tr}\left\{I_{x}\hat{T}e^{-i\int_{0}^{t_{\text{SL}}}dtH_{\text{total}}^{\prime }}I_{x}\hat{T}e^{i\int_{0}^{t_{\text{SL}}}dtH_{\text{total}}^{\prime }}\right\}\;,$$

where $\hat{T}$ is a Dyson operator and $H_{\text{total}}^{\prime }$ is a total Hamiltonian. We consider the effects of $B_{0}$ and $B_{1}$ modulations or dipolar interaction. For all three sources, $H_{\text{total}}^{\prime }$ can be defined as follows:

2 $$\begin{array}{rl} H_{\text{total}}^{\prime }= & H_{\text{SL}}^{\prime }+H_{t}^{\prime }=\omega_{\text{SL}}I_{x}+2\pi \sum_{n}a_{n}\mathrm{cos}(n\omega_{R}t+\phi_{n})\\ & \times [I_{z}\mathrm{cos}\varphi +I_{y}\mathrm{sin}\varphi ]\hat{\text{Op}}\;, \end{array}$$

where $\omega_{\text{SL}}=2\pi \nu_{\text{SL}}$ and $H_{\text{SL}}^{\prime }$ is an ideal spin-lock Hamiltonian. Here, $\hat{\text{Op}}=1$ for a single spin with $B_{0}$ ( φ≥0 ) or $B_{1}$ ( φ=π/2 ) modulations or $\hat{\text{Op}}=2S_{z}$ with φ=0 for a two-spin system (dipolar interaction). While for dipolar interaction, n is 1 or 2 (Mehring, 1983; Olejniczak et al., 1984), for $B_{0}$ and $B_{1}$ modulations, n may take any integer value (Aebischer et al., 2021). This is because these modulations are not purely sinusoidal; there are contributions from overtone frequencies. In the experimental SL profiles (Figs. 2 and 3), two rotary resonance conditions are clearly observed. Therefore, in the following discussion, n=1,2 will be considered for all three cases. Also note that for the cosine modulated terms of Eq. (2), only $I_{y}$ (and not $I_{z}$ ) survives the rotating frame transformation and secular approximation for the case of $B_{1}$ modulation. Both terms are relevant for $B_{0}$ modulations. For the dipolar interaction, $a_{n}$ inversely depend on the distance between the pair of spins and the orientation (Mehring, 1983; Olejniczak et al., 1984): $a_{1}=\nu_{D}/\sqrt{2}\mathrm{sin}(2\beta )$ and $a_{2}=-\nu_{D}/2{\mathrm{sin}}^{2}(\beta )$ ; $\nu_{D}=\nu_{\text{D,IS}}=-\mu_{0}/(8\pi^{2})(\hbar \gamma_{I}\gamma_{S})/(r_{\text{IS}}^{3})$ and (β) is the Euler angle with respect to the rotor frame (Mehring, 1983). For $B_{0}$ and $B_{1}$ modulations, $a_{n}$ values do not exhibit any orientation dependence. It is worth noting that for $B_{1}$ modulations, $a_{n}$ values change with the strength of the applied RF-field lock value ( $\nu_{\text{SL}}$ ).

If φ does not vary with time, Eq. (2) can be simplified by rotation of $H_{\text{total}}^{\prime }$ by an φ angle around the $\hat{x}$ using the operator $e^{i\varphi I_{x}}$ . Such a rotation removes any dependence on φ , since the initial and the final operators in Eq. (1) commute with $e^{i\varphi I_{x}}$ . The modified version of Eq. (2) is written as follows:

3 $$\begin{array}{rl} H_{\text{total}}= & e^{-i\varphi I_{x}}H_{\text{total}}^{\prime }e^{i\varphi I_{x}}=H_{\text{SL}}+H_{t}\\ = & \omega_{\text{SL}}I_{x}+2\pi \sum_{n}a_{n}\mathrm{cos}(n\omega_{R}t+\phi_{n})I_{z}\hat{\text{Op}}\;. \end{array}$$

Thus, while $B_{0}$ modulation may occur anywhere in the y – z plane, the theoretical treatment remains exactly the same as for z modulation. Mathematically, this is also true for $B_{1}$ modulation, while physically, these modulations are only relevant when in the transverse plane.

In the Supplement, using average Hamiltonian theory (AHT) and considering only the first-order terms (Haeberlen and Waugh, 1968) under rotary resonance conditions ( $\nu_{\text{SL}}=\nu_{R}$ or $2\nu_{R}$ ), the measured SL signal for $B_{0}$ or $B_{1}$ modulations is as follows:

4 $$S_{\text{SL}}(t_{\text{SL}}=N_{\text{SL}}T_{R})\approx \mathrm{cos}(\pi a_{k}t_{\text{SL}})\;,$$

while for dipolar interaction,

5 $$S_{\text{SL}}(t_{\text{SL}}=N_{\text{SL}}T_{R})\approx \int d\Omega \mathrm{cos}(\pi a_{k}t_{\text{SL}})\;,$$

where the integration over orientation ( Ω ) indicates the powder averaging with Euler angles, (α,β,γ) (Mehring, 1983) and k = 1 or 2. The derivations of Eqs. (4) and (5) are shown in Eqs. (S1)–(S11) in the Supplement.

**Figure 4:**
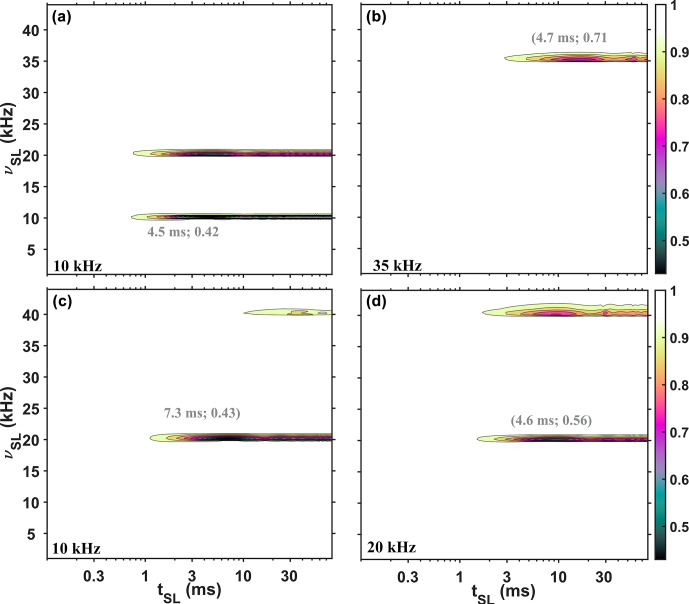
Simulated SL profiles showing the influence of time dependence introduced via $B_{0}$ modulation, including distributions in SL frequency and in amplitude of $B_{0}$ modulation. The simulated signal is shown as a function of the RF-field strength ( $\nu_{\text{SL}}$ , axis y ) and mixing time ( $t_{\text{SL}}$ , axis x ) of the SL under three different MAS rates: 10 kHz **(a)** and **(c)**, 20 kHz **(d)**, and 35 kHz **(b)**. For **(a)**, **(b)**, and **(d)**, continuous SL was applied, while for **(c)** windowed SL was implemented (half of the rotor period was filled with the pulse). The values in gray represent the coordinates of the first minima in the profiles. No phenomenological relaxation was included in the simulations. Additional simulated details are provided in the Supplement.

**Figure 5:**
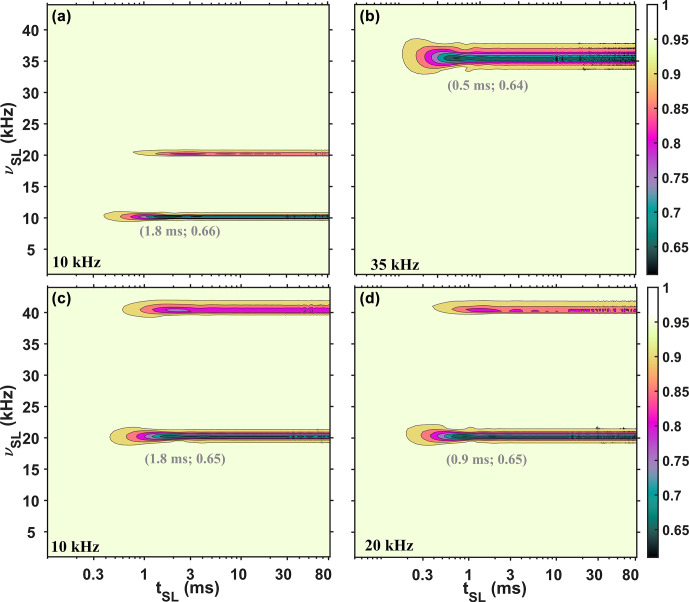
Simulated SL profiles showing the influence of time dependence introduced via $B_{1}$ modulation, including distributions in the SL frequency and in the amplitude of $B_{1}$ modulation. The simulated signal is shown as functions of the RF-field strength ( $\nu_{\text{SL}}$ , axis y ) and mixing time ( $t_{\text{SL}}$ , axis x ) of the SL under three different MAS rates: 10 kHz **(a)** and **(c)**, 20 kHz **(d)**, and 35 kHz **(b)**. For **(a)**, **(b)**, and **(d)**, continuous SL was applied, while for **(c)** windowed (half of the rotor period was filled with the pulse) SL was implemented. The values in gray represent the coordinates of the first minima points in the profiles. Relaxation was not included in the simulations. Additional simulated details are provided in the Supplement.

The complete agreement between AHT and numerical simulations of SL signals (Figs. S3 and S4 in the Supplement) indicates that this effect is fully explained with first-order terms. Note that the simulations are fully coherent in origin. The change in MAS rate affects only $B_{1}$ -induced signal modulations (Figs. S4 and S5 in the Supplement), since the $B_{1}$ field is also increased at the resonance condition. Specifically, the strength of field oscillations ( $a_{k}$ ) increases linearly with the $B_{1}$ field, which matches the MAS frequency at the resonance condition, and therefore the signal modulation frequency also increases linearly. In the case of $B_{0}$ modulation, adjustments to the shimming coil are expected to have a profound effect, but oscillations in signal amplitude are expected to be independent of the applied $B_{1}$ field. By contrast, for $B_{1}$ modulation, changes in the strength of the applied spin lock have a major effect, since the oscillation frequency of signal amplitude is expected to depend on $B_{1}$ . These observations already point to $B_{1}$ as the most likely source of the observed pseudo-RRD effect, since the position of the first signal minimum was observed to profoundly depend on the MAS frequency (Fig. 2).

A better match between experiments and simulations logically requires consideration of distributions in various parameters representing the position dependence of the sample. Based on Fig. S5, for all three sources, the rotary resonance conditions are very narrow. However, the addition of a spatial distribution of applied $\nu_{\text{SL}}$ values to $H_{\text{SL}}$ broadens these conditions (Eq. S14 and Fig. S6 in the Supplement), making them more experimentally detectable and damping oscillations.

More generally, it makes sense to also consider distributions in the amplitude of $B_{0}$ or $B_{1}$ modulations (Eqs. S15 and S16 in the Supplement). The specific spatial distributions chosen for $B_{0}$ and $B_{1}$ are summarized in Table S1 in the Supplement and shown in Figs. S7 and S8 in the Supplement. The types of inhomogeneity used roughly match the expectation for solenoidal coils, where the sample near the ends of the coil experiences a lower RF-field strength. Figures 4 and 5 show simulations for $B_{0}$ and $B_{1}$ modulation that include these distributions. The inclusion of distributions in the simulation primarily broadens the rotary resonance conditions and affects the frequency and amplitude of the modulations in the spin-lock signals. Relatively good agreement is observed between the experiment and simulation despite the imprecise simulation of a spatial distribution of $B_{1}$ . A more quantitative assessment would call for calculation of the exact values and shapes of $B_{1}$ fields for a particular coil, as well as better characterization of $B_{0}$ distributions (Aebischer et al., 2021; Engelke, 2002; Guenneugues et al., 1999; Hürlimann and Griffin, 2000; Lips et al., 2001; Odedra and Wimperis, 2013; Paulson et al., 2004; Privalov et al., 1996; Schönzart et al., 2024; Tošner et al., 2017, 2018). Note that the distributions are reasonable, considering the published calculations for solenoidal coils (Gupta et al., 2015; Tošner et al., 2017; Uribe et al., 2024).

Figure 4 shows simulations for $B_{0}$ modulation that include distributions in the SL frequency and in the amplitude of $B_{0}$ modulation. While some similarities are seen compared with the experimental data (Fig. 2), there are three major differences in the SL profiles, which should be highlighted. Firstly, in Fig. 4, the intensities at the first minima show a dependence on MAS rate (marked in gray in Fig. 4), whereas in Fig. 2, the experimental profiles show only a slight dependence. Secondly, in Fig. 4, the locations of these minima in time ( x axis) do not depend on the MAS rate (Fig. 4a, b, and d) but are different when windowed pulses are applied (Fig. 4c). In contrast, the experimental profiles exhibit the reverse behavior. Thirdly, with windowed pulses, as in Fig. 4c, the second rotary resonance condition is attenuated compared to continuous spin lock, while in Fig. 2c two rotary resonance conditions are clearly detected. Additionally, increasing the magnetic field inhomogeneity by deliberately mis-setting the room temperature shims had little influence on the SL profile (shown in Fig. S2 in the Supplement).

All of this indicates that a $B_{0}$ modulation cannot be a major source of the appearance of rotary resonance conditions in these rotating liquids and liquid-like samples.

In contrast, simulations of SL profiles with time dependence introduced via $B_{1}$ modulation (Fig. 5) qualitatively agree with the experimental plots, indicating that a $B_{1}$ modulation is a better explanation for the appearance of rotary resonance conditions in rotating liquids and liquid-like samples using conventional MAS NMR probes with solenoidal coils. Hardware limitations including such time dependence have been considered previously in the design of magnetization transfer elements using optimal control (Blahut et al., 2022, 2023; Glaser et al., 2015; Joseph and Griesinger, 2023; Tošner et al., 2017, 2018).

This qualitative explanation, provided by simulations, indicates that this effect can also be anticipated in experiments involving solid samples, in addition to the desired effects caused by molecular motion. It is therefore recommended to consider coil inhomogeneity when measuring relaxation rates near rotary resonance conditions. Fortunately, the magnitude of this effect is considerably smaller than the strong relaxation observed in recent reports that detected slow structural dynamics via near rotary resonance conditions (Krushelnitsky et al., 2018).

## 3 Conclusions

Rotary resonance conditions, under which applied RF-field strength equals an even multiple of the MAS rate, provide a powerful avenue to obtain specific structural information via recoupling of anisotropic interactions in solids (De Paëpe, 2012; Nishiyama et al., 2022) or for detecting changes in the relaxation rates due to slow motion in the microsecond ( µs ) range (Rovó, 2020). Canonically, rotary resonance conditions are not expected in liquids due to the averaging of first-order anisotropic interactions from (sub)nanosecond isotropic motion (Haeberlen and Waugh, 1968; Maricq, 1982). In this article, we present experimental data, in which we detected rotary resonance conditions in a liquid and a liquid-like sample. We qualitatively explain the major source of these conditions, which can occur from a combination of two factors: the rotation of the sample and a spatially inhomogeneous RF field generated by a solenoidal coil (Tošner et al., 2017). As a result, the RF-field Hamiltonian contains time-dependent terms, which leads to signal decrease, i.e., pseudo relaxation behavior, at or near rotary resonance conditions. To mitigate these effects, it may be advantageous to consider different hardware designs (Chen et al., 2018; Xu et al., 2021), e.g., RF coils that produce more homogeneous RF fields (Grant et al., 2010; Kelz et al., 2019; Krahn et al., 2008; Stringer et al., 2005)

## Supplement

10.5194/mr-6-119-2025-supplement The supplement related to this article is available online at https://doi.org/10.5194/mr-6-119-2025-supplement.

## Supplement

10.5194/mr-6-119-2025-supplement
10.5194/mr-6-119-2025-supplement The supplement related to this article is available online at https://doi.org/10.5194/mr-6-119-2025-supplement.

## Data Availability

The NMR datasets are available from Zenodo at https://doi.org/10.5281/zenodo.15478341 (Nimerovsky, 2025).

## Code availability

For the simulations, in-house MATLAB scripts were used. The scripts are available as part of the Supplement and are located in the MATLAB_codes folder.

## Appendix

The supplement related to this article is available online at https://doi.org/10.5194/mr-6-119-2025-supplement.

## References

[bib1.bib1] Abyzov A, Blackledge M, Zweckstetter M (2022). Conformational Dynamics of Intrinsically Disordered Proteins Regulate Biomolecular Condensate Chemistry. Chem Rev.

[bib1.bib2] Aebischer K, Tošner Z, Ernst M (2021). Effects of radial radio-frequency field inhomogeneity on MAS solid-state NMR experiments. Magn Reson.

[bib1.bib3] Alam MK, Bhuvaneshwari RA, Sengupta I (2024). ${}^{19}F$
 NMR relaxation of buried tryptophan side chains suggest anisotropic rotational diffusion of the protein RfaH. J Biomol NMR.

[bib1.bib4] Andrew ER, Bradbury A, Eades RG (1958). Nuclear Magnetic Resonance Spectra from a Crystal rotated at High Speed. Nature.

[bib1.bib5] Blahut J, Brandl MJ, Pradhan T, Reif B, Tošner Z (2022). Sensitivity-Enhanced Multidimensional Solid-State NMR Spectroscopy by Optimal-Control-Based Transverse Mixing Sequences. J Am Chem Soc.

[bib1.bib6] Blahut J, Brandl MJ, Sarkar R, Reif B, Tošner Z (2023). Optimal control derived sensitivity-enhanced CA-CO mixing sequences for MAS solid-state NMR – Applications in sequential protein backbone assignments. J Magn Reson Open.

[bib1.bib7] Camacho-Zarco AR, Schnapka V, Guseva S, Abyzov A, Adamski W, Milles S, Jensen MR, Zidek L, Salvi N, Blackledge M (2022). NMR Provides Unique Insight into the Functional Dynamics and Interactions of Intrinsically Disordered Proteins. Chem Rev.

[bib1.bib8] Cavanagh J, Fairbrother WJ, Palmer III AG, Rance M, Skelton NJ (2006). Protein NMR Spectroscopy: Principles and Practice.

[bib1.bib9] Chen P, Albert BJ, Gao C, Alaniva N, Price LE, Scott FJ, Saliba EP, Sesti EL, Judge PT, Fisher EW, Barnes AB (2018). Magic angle spinning spheres. Sci Adv.

[bib1.bib10] Cohen-Addad JP, Vogin R (1974). Molecular Motion Anisotropy as Reflected by a “Pseudosolid” Nuclear Spin Echo: Observation of Chain Constraints in Molten *cis*-1,4-Polybutadiene. Phys Rev Lett.

[bib1.bib11] De Paëpe G (2012). Dipolar Recoupling in Magic Angle Spinning Solid-State Nuclear Magnetic Resonance. Annu Rev Phys Chem.

[bib1.bib12] Deverell C, Morgan RE, Strange JH (1970). Studies of chemical exchange by nuclear magnetic relaxation in the rotating frame. Mol Phys.

[bib1.bib13] Engelke F (2002). Electromagnetic wave compression and radio frequency homogeneity in NMR solenoidal coils: Computational approach. Concepts Magn Reso.

[bib1.bib14] Fonseca R, Vieira R, Sardo M, Marin-Montesinos I, Mafra L (2022). Exploring Molecular Dynamics of Adsorbed 
${\mathrm{CO}}_{2}$
 Species in Amine-Modified Porous Silica by Solid-State NMR Relaxation. J Phys Chem C.

[bib1.bib15] Furman GB, Panich AM, Goren SD (1998). Spin-locking in one pulse NMR experiment. Solid State Nucl Mag.

[bib1.bib16] Glaser SJ, Boscain U, Calarco T, Koch CP, Köckenberger W, Kosloff R, Kuprov I, Luy B, Schirmer S, Schulte-Herbrüggen T, Sugny D, Wilhelm FK (2015). Training Schrödinger's cat: quantum optimal control. Eur Phys J D.

[bib1.bib17] Goldman M, Tekely P (2001). Effect of radial RF field on MAS spectra. CR Acad Sci II C.

[bib1.bib18] Grant CV, Wu CH, Opella SJ (2010). Probes for high field solid-state NMR of lossy biological samples. J Magn Reson.

[bib1.bib19] Guenneugues M, Berthault P, Desvaux H (1999). A Method for Determining 
B1
 Field Inhomogeneity. Are the Biases Assumed in Heteronuclear Relaxation Experiments Usually Underestimated?. J Magn Reson.

[bib1.bib20] Gupta R, Hou G, Polenova T, Vega A J (2015). RF Inhomogeneity and how it Control CPMAS. Solid State Nucl Magn Reson.

[bib1.bib21] Haeberlen U, Waugh JS (1968). Coherent Averaging Effects in Magnetic Resonance. Phys Rev.

[bib1.bib22] Hahn EL (1950). Spin Echoes. Phys Rev.

[bib1.bib23] Hartmann SR, Hahn EL (1962). Nuclear Double Resonance in the Rotating Frame. Phys Rev.

[bib1.bib24] Hoult DI (1976). Solvent peak saturation with single phase and quadrature fourier transformation. J Magn Reson.

[bib1.bib25] Hu Y, Cheng K, He L, Zhang X, Jiang B, Jiang L, Li C, Wang G, Yang Y, Liu M (2021). NMR-Based Methods for Protein Analysis. Anal Chem.

[bib1.bib26] Hürlimann MD, Griffin DD (2000). Spin Dynamics of Carr–Purcell–Meiboom–Gill-like Sequences in Grossly Inhomogeneous 
$B_{0}$
 and 
$B_{1}$
 Fields and Application to NMR Well Logging. J Magn Reson.

[bib1.bib27] Joseph D, Griesinger C (2023). Optimal control pulses for the 1.2 GHz (28.2-T) NMR spectrometers. Sci Adv.

[bib1.bib28] Keeler EG, McDermott AE (2022). Rotating Frame Relaxation in Magic Angle Spinning Solid State NMR, a Promising Tool for Characterizing Biopolymer Motion. Chem Rev.

[bib1.bib29] Kelz JI, Kelly JE, Martin RW (2019). 3D-printed dissolvable inserts for efficient and customizable fabrication of NMR transceiver coils. J Magn Reson.

[bib1.bib30] Krahn A, Priller U, Emsley L, Engelke F (2008). Resonator with reduced sample heating and increased homogeneity for solid-state NMR. J Magn Reson.

[bib1.bib31] Krushelnitsky A, Gauto D, Rodriguez Camargo DC, Schanda P, Saalwächter K (2018). Microsecond motions probed by near-rotary-resonance 
R1ρ


${}^{15}N$
 MAS NMR experiments: the model case of protein overall-rocking in crystals. J Biomol NMR.

[bib1.bib32] Krushelnitsky A, Hempel G, Jurack H, Mendes Ferreira T (2023). Rocking motion in solid proteins studied by the 
${}^{15}N$
 proton-decoupled 
R1ρ
 relaxometry. Phys Chem Chem Phys.

[bib1.bib33] Kupče Ē, Keifer PA, Delepierre M (2001). Adiabatic TOCSY MAS in Liquids. J Magn Reson.

[bib1.bib34] Kurauskas V, Izmailov SA, Rogacheva ON, Hessel A, Ayala I, Woodhouse J, Shilova A, Xue Y, Yuwen T, Coquelle N, Colletier J-P, Skrynnikov NR, Schanda P (2017). Slow conformational exchange and overall rocking motion in ubiquitin protein crystals. Nat Commun.

[bib1.bib35] Kurbanov R, Zinkevich T, Krushelnitsky A (2011). The nuclear magnetic resonance relaxation data analysis in solids: General 
R1/R1ρ
 equations and the model-free approach. J Chem Phys.

[bib1.bib36] Lee YK, Kurur ND, Helmle M, Johannessen OG, Nielsen NC, Levitt MH (1995). Efficient dipolar recoupling in the NMR of rotating solids. A sevenfold symmetric radiofrequency pulse sequence. Chem Phys Lett.

[bib1.bib37] Levitt MH, Oas TG, Griffin RG (1988). Rotary Resonance Recoupling in Heteronuclear Spin Pair Systems. Isr J Chem.

[bib1.bib38] Lewandowski JR, Sass HJ, Grzesiek S, Blackledge M, Emsley L (2011). Site-Specific Measurement of Slow Motions in Proteins. J Am Chem Soc.

[bib1.bib39] Lips O, Privalov AF, Dvinskikh SV, Fujara F (2001). Magnet Design with High 
B0
 Homogeneity for Fast-Field-Cycling NMR Applications. J Magn Reson.

[bib1.bib40] Lowe IJ (1959). Free Induction Decays of Rotating Solids. Phys Rev Lett.

[bib1.bib41] Ma P, Haller JD, Zajakala J, Macek P, Sivertsen A C, Willbold D, Boisbouvier J, Schanda P (2014). Probing Transient Conformational States of Proteins by Solid-State 
R1ρ
 Relaxation-Dispersion NMR Spectroscopy. Angew Chem Int Edit.

[bib1.bib42] Malinowski ER, Pierpaoli AR (1969). Asymmetric spinning sidebands from coaxial cells in NMR spectra. J Magn Reson.

[bib1.bib43] Maricq MM (1982). Application of average Hamiltonian theory to the NMR of solids. Phys Rev B.

[bib1.bib44] Marion D, Gauto DF, Ayala I, Giandoreggio-Barranco K, Schanda P (2019). Microsecond Protein Dynamics from Combined Bloch-McConnell and Near-Rotary-Resonance R1 Relaxation-Dispersion MAS NMR. Chem Phys Chem.

[bib1.bib45] Massi F, Peng JW, Ghose R (2018). Protein NMR: Methods and Protocols.

[bib1.bib46] Mehring M (1983). Principles of High Resolution NMR in Solids.

[bib1.bib47] Nimerovsky E (2025). Zenodo.

[bib1.bib48] Nimerovsky E, Goldbourt A (2012). Insights into the spin dynamics of a large anisotropy spin subjected to long-pulse irradiation under a modified REDOR experiment. J Magn Reson.

[bib1.bib49] Nimerovsky E, Becker S, Andreas LB (2023). Windowed cross polarization at 55 kHz magic-angle spinning. J Magn Reson.

[bib1.bib50] Nishiyama Y, Hou G, Agarwal V, Su Y, Ramamoorthy A (2022). Ultrafast Magic Angle Spinning Solid-State NMR Spectroscopy: Advances in Methodology and Applications. Chem Rev.

[bib1.bib51] Oas TG, Griffin RG, Levitt MH (1988). Rotary resonance recoupling of dipolar interactions in solid-state nuclear magnetic resonance spectroscopy. J Chem Phys.

[bib1.bib52] Odedra S, Wimperis S (2013). Imaging of the 
B
1 distribution and background signal in a MAS NMR probehead using inhomogeneous 
$B_{0}$
 and 
$B_{1}$
 fields. J Magn Reson.

[bib1.bib53] Olejniczak ET, Vega S, Griffin RG (1984). Multiple pulse NMR in rotating solids. J Chem Phys.

[bib1.bib54] Öster C, Kosol S, Lewandowski JR (2019). Quantifying Microsecond Exchange in Large Protein Complexes with Accelerated Relaxation Dispersion Experiments in the Solid State. Sci Rep-UK.

[bib1.bib55] Palmer AG, Massi F (2006). Characterization of the Dynamics of Biomacromolecules Using Rotating-Frame Spin Relaxation NMR Spectroscopy. Chem Rev.

[bib1.bib56] Palmer AGI (2004). NMR Characterization of the Dynamics of Biomacromolecules. Chem Rev.

[bib1.bib57] Palmer AGI (2015). Enzyme Dynamics from NMR Spectroscopy. Acc Chem Res.

[bib1.bib58] Paulson EK, Martin RW, Zilm KW (2004). Cross polarization, radio frequency field homogeneity, and circuit balancing in high field solid state NMR probes. J Magn Reson.

[bib1.bib59] Pratihar S, Sabo TM, Ban D, Fenwick RB, Becker S, Salvatella X, Griesinger C, Lee D (2016). Kinetics of the Antibody Recognition Site in the Third IgG-Binding Domain of Protein G. Angew Chem Int Edit.

[bib1.bib60] Privalov AF, Dvinskikh SV, Vieth H-M (1996). Coil Design for Large-Volume High-
$B_{1}$
 Homogeneity for Solid-State NMR Applications. J Magn Reson Ser A.

[bib1.bib61] Quinn CM, McDermott AE (2009). Monitoring conformational dynamics with solid-state 
R1ρ
 experiments. J Biomol NMR.

[bib1.bib62] Rangadurai A, Szymaski ES, Kimsey IJ, Shi H, Al-Hashimi HM (2019). Characterizing micro-to-millisecond chemical exchange in nucleic acids using off-resonance 
R1ρ
 relaxation dispersion. Prog Nucl Mag Res Sp.

[bib1.bib63] Redfield AG (1957). On the Theory of Relaxation Processes. IBM J Res Dev.

[bib1.bib64] Rovó P (2020). Recent advances in solid-state relaxation dispersion techniques. Solid State Nucl Magn Reson.

[bib1.bib65] Rovó P, Linser R (2018). Microsecond Timescale Protein Dynamics: a Combined Solid-State NMR Approach. Chem Phys Chem.

[bib1.bib66] Schanda P, Ernst M (2016). Studying dynamics by magic-angle spinning solid-state NMR spectroscopy: Principles and applications to biomolecules. Prog Nucl Magn Res Sp.

[bib1.bib67] Schmidt-Rohr K, Clauss J, Spiess HW (1992). Correlation of structure, mobility, and morphological information in heterogeneous polymer materials by two-dimensional wideline-separation NMR spectroscopy. Macromolecules.

[bib1.bib68] Schönzart J, Han R, Gennett T, Rienstra CM, Stringer JA (2024). Magnetic Susceptibility Modeling of Magic-Angle Spinning Modules for Part Per Billion Scale Field Homogeneity. J Magn Reson.

[bib1.bib69] Sekhar A, Kay LE (2019). An NMR View of Protein Dynamics in Health and Disease. Annu Rev Biophys.

[bib1.bib70] Shaka AJ, Keeler J, Frenkiel T, Freeman R (1983). An improved sequence for broadband decoupling: WALTZ-16. J Magn Reson.

[bib1.bib71] Shcherbakov AA, Brousseau M, Henzler-Wildman KA, Hong M (2023). Microsecond Motion of the Bacterial Transporter EmrE in Lipid Bilayers. J Am Chem Soc.

[bib1.bib72] Sodickson A, Cory DG (1997). Shimming a High-Resolution MAS Probe. J Magn Reson.

[bib1.bib73] Stief T, Vormann K, Lakomek N-A (2024). Sensitivity-enhanced NMR 
${}^{15}N$


R1
 and 
R1ρ
 relaxation experiments for the investigation of intrinsically disordered proteins at high magnetic fields. Methods.

[bib1.bib74] Stringer JA, Bronnimann CE, Mullen CG, Zhou DH, Stellfox SA, Li Y, Williams EH, Rienstra CM (2005). Reduction of RF-induced sample heating with a scroll coil resonator structure for solid-state NMR probes. J Magn Reson.

[bib1.bib75] Tekely P, Goldman M (2001). Radial-Field Sidebands in MAS. J Magn Reson.

[bib1.bib76] Tošner Z, Purea A, Struppe JO, Wegner S, Engelke F, Glaser SJ, Reif B (2017). Radiofrequency fields in MAS solid state NMR probes. J Magn Reson.

[bib1.bib77] Tošner Z, Sarkar R, Becker-Baldus J, Glaubitz C, Wegner S, Engelke F, Glaser SJ, Reif B (2018). Overcoming Volume Selectivity of Dipolar Recoupling in Biological Solid-State NMR Spectroscopy. Angew Chem Int Edit.

[bib1.bib78] Uribe JL, Jimenez MD, Kelz JI, Liang J, Martin RW (2024). Automated test apparatus for bench-testing the magnetic field homogeneity of NMR transceiver coils. J Magn Reson Open.

[bib1.bib79] Vera M, Grutzner JB (1986). The Taylor vortex: the measurement of viscosity in NMR samples. J Am Chem Soc.

[bib1.bib80] Vold RL, Waugh JS, Klein MP, Phelps DE (1968). Measurement of Spin Relaxation in Complex Systems. J Chem Phys.

[bib1.bib81] Vugmeyster L, Ostrovsky D, Greenwood A, Fu R (2022). Deuteron rotating frame relaxation for the detection of slow motions in rotating solids. J Magn Reson.

[bib1.bib82] Vugmeyster L, Rodgers A, Ostrovsky D, McKnight JC, Fu R (2023). Deuteron off-resonance rotating frame relaxation for the characterization of slow motions in rotating and static solid-state proteins. J Magn Reson.

[bib1.bib83] Wang AC, Bax A (1993). Minimizing the effects of radio-frequency heating in multidimensional NMR experiments. J Biomol NMR.

[bib1.bib84] Xu K, Pecher O, Braun M, Schmedt auf der Günne J (2021). Stable magic angle spinning with Low-Cost 3D-Printed parts. J Magn Reson.

[bib1.bib85] Zektzer AS, Swanson MG, Jarso S, Nelson SJ, Vigneron DB, Kurhanewicz J (2005). Improved signal to noise in high-resolution magic angle spinning total correlation spectroscopy studies of prostate tissues using rotor-synchronized adiabatic pulses. Magn Reson Med.

