# Supplementary material for: Pseudo rotary resonance relaxation dispersion effects in isotropic samples
Source: Magn Reson (Gott). 2025 Jun 3;6(1):119–29. doi: 10.5194/mr-6-119-2025 (PMC12247080; doi:10.5194/mr-6-119-2025)
Supplement: The supplement related to this article is available online at https://doi.org/10.5194/mr-6-119-2025-supplement. [file mr-6-119-2025-supplement.zip › pseudoRRD_revII_SI.pdf]

Pseudo Rotary Resonance Relaxation Dispersion Effects in Isotropic Samples

Authors: *Evgeny Nimerovsky*, Jonas Mehrens & Loren B. Andreas**

Affiliations:

Department of NMR based Structural Biology, Max Planck Institute for Multidisciplinary Sciences, Am Faßberg 11, Göttingen, Germany

*Corresponding authors: land@mpinat.mpg.de ORCID: 0000-0003-3216-9065 and evni@mpinat.mpg.de ORCID: 0000-0003-3002-0718.

Additional Experiments

Figure S1A shows the comparison between experimental spin-echo curve(Hahn, 1950) (stars) and mono-exponential fitting (black line) with the obtained T_2 value of 88 ms from least-square fitting. Comparison of the experimental spin-lock curves with continuous (red triangles) and half-rotor-filled (blue squares) rf-field pulses show that the $T_{2\rho}$ value remains unchanged.

Figure S1B shows the comparison between experimental inversion recovery curve(Vold et al., 1968) (stars) and mono-exponential fitting (black line) with the obtained T_1 value of 680 ms from least-square fitting.

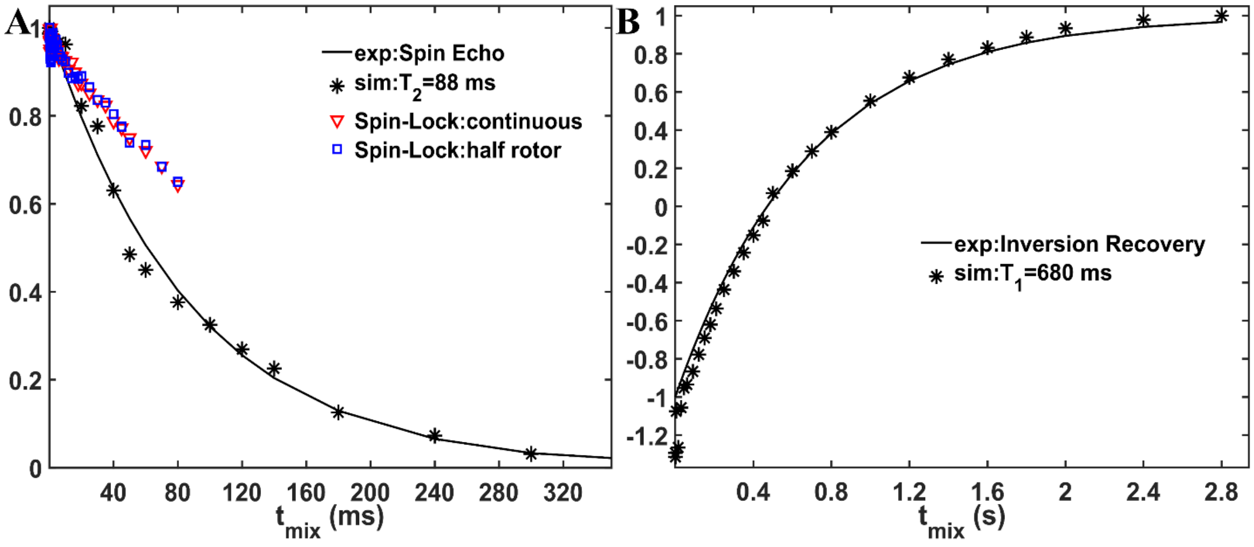

Figure S1 ^{13}C T2 / T1ρ (A) and T1 (B) measurement for polybutadiene rubber. (A) The experimental spin-echo (stars) and mono-exponential fit (black line) at 20 kHz MAS. The red triangles and blue squares represent experimental spin-lock curves with continuous (at 10 kHz MAS) and half-rotor-filled (at 20 kHz MAS) rf-field pulses, respectively. For each case, 1 kHz rf-field strength was applied. (B) The experimental inversion recovery (stars) and mono-exponential (black line) curves at 20 kHz MAS.

Figure S2A shows the 1D ^{13}C spectrum of polybutadiene rubber at 10 kHz MAS (black) and without spinning (red). Figure S2B shows the 1D ^{13}C spectrum of polybutadiene rubber at 10 kHz MAS with optimal (black) and deliberately degraded (red) shimming files. Figure S2C-E shows SL profiles with optimal shimming (C, same as Figure 2A) and deliberately degraded (D and E) shimming files. The same data is shown was used for D and E, and displayed either as intensities (D) or integrals of the signal (E).

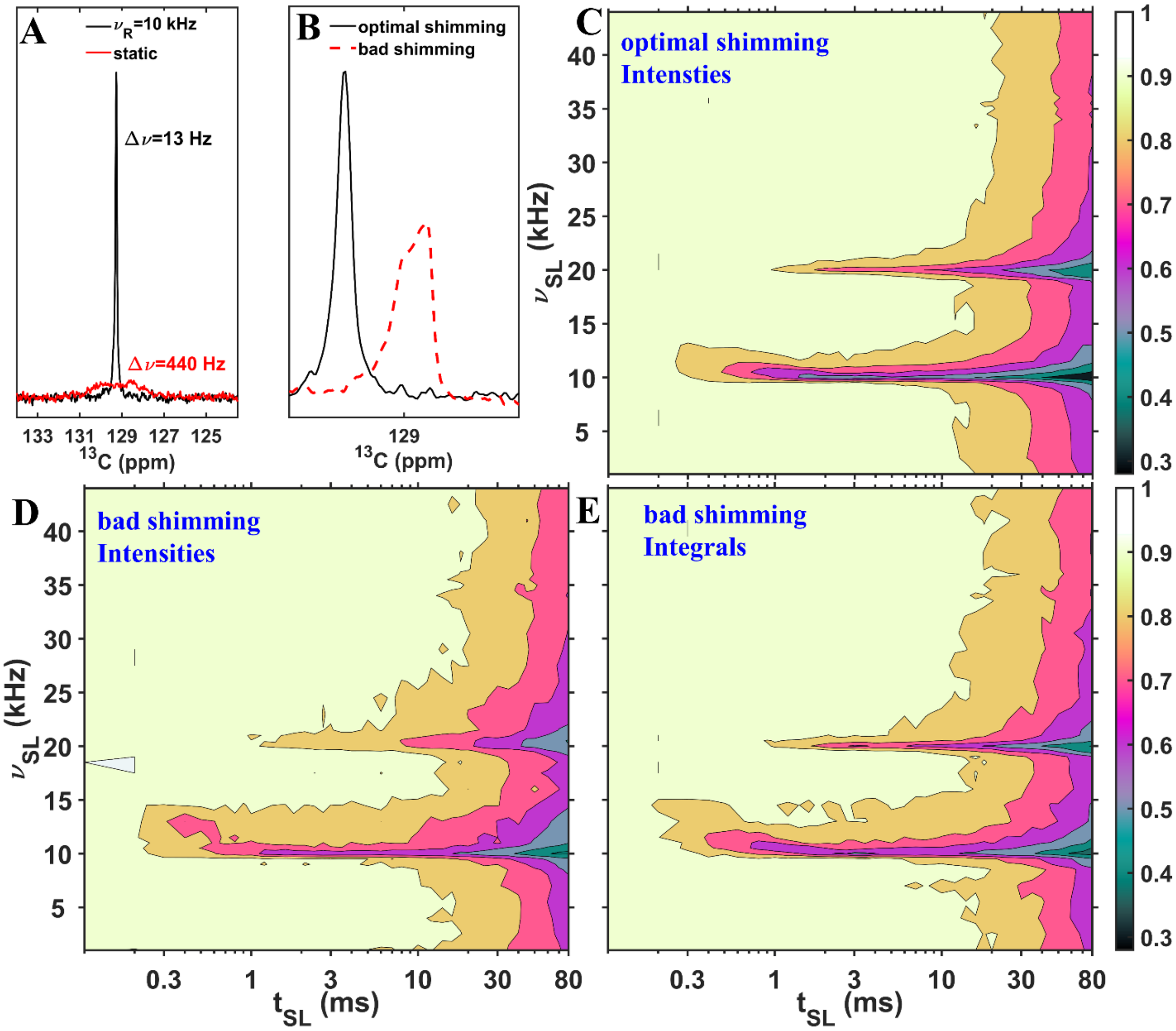

Figure S2 ^{13}C spectra and SL profiles of polybutadiene rubber with different shims. (A) 1D signal at 10 kHz MAS (black) and static (red). (B) 1D signal at 10 kHz MAS with optimal (black) and deliberately degraded (red) shimming. (C-E) ^{13}C signal is shown as a functions of the rf-field strength (ν_{SL}, y-axis) and mixing time (t_{SL}, x-axis) of the SL with optimal shims (C) and deliberately degraded shims (D and E). For (D) and (E), the same acquired data was used; the difference is that in (D), the intensity is displayed, while in (E), the signal integral is shown.

Additional simulations and FOH

To understand the origin of the pseudo-RRD effect, we start with the simplest case, investigating the behavior of an on-resonance spin (I) during the rf-field spin-lock (H_{SL}). The

single spin inside the coil may be affected by an additional time-periodic term (H_t), orthogonal to the applied rf-field spin-lock. For simplicity, we also do not include any relaxation effects.

This additional term can depend on the external magnetic field (B_0 modulation), or the strength of the applied RF-field spin-lock (B_1 modulation). In solid samples, it can arise due to anisotropic interactions. The first and second modulations are related to inhomogeneities in the external magnetic field and RF field, respectively. The third could arise in the samples if there is some degree of alignment and therefore residual anisotropic interactions present. Knowing the exact values of these modulations (and shapes in the case of the distribution) is important when their influence is investigated quantitatively for a specific coil. Our goal here is the qualitative determination of the source of the pseudo-RRD effect in the experiments.

In all three cases, the total Hamiltonian for this spin (starting from Eqn. (3) in the main text) can be described as follows:

$$H_{total} = H_{SL} + H_t = \omega_{SL} I_x + 2\pi \sum_n a_n \cos(n\omega_R t + \phi_n) I_z \widehat{Op}, \qquad \text{Eqn. (S1)}$$

where $\omega_{SL} = 2\pi\nu_{SL}$. Here, $\widehat{Op} = 1$ for a single spin or $\widehat{Op} = 2S_z$ for a two-spin system. While for anisotropic interactions, n is 1 or 2,(Mehring, 1983; Olejniczak et al., 1984) for B_0 and B_1 modulations, n may take any integer value.(Aebischer et al., 2021) This is because these modulations are not purely sinusoidal, there are contributions from overtone frequencies. In the experimental SL profiles (Figures 2 and 3 in the main text), two rotary-resonance conditions are clearly observed. Therefore, in the following discussion, $n = 1, 2$ will be considered for all three cases.

The simulated SL-signal is defined as follows:

$$S_{SL}(t_{SL}) = \mathrm{Tr}\left\{I_x \widehat{T} e^{-i\int_0^{t_{SL}} dt H_{total}} I_x \widehat{T} e^{i\int_0^{t_{SL}} dt H_{total}}\right\}, \quad \text{Eqn. (S2)}$$

where $\widehat{T}$ is a Dyson operator. To simplify Eqn. (S1), the total Hamiltonian is transformed into the tilted rf-field frame:(Mehring, 1983)

$$H_{tot}^{rf} = U_{SL}^{-1} H_{tot}(t) U_{SL} - H_{SL}, \quad \text{Eqn. (S3)}$$

where $U_{SL} = e^{-i\omega_{SL} t I_x}$ is a propagator. The modified Eqn. (S2) in the titled frame is written as follows:

$$S_{SL}(t_{SL}) = \mathrm{Tr}\left\{I_x \widehat{T} e^{-i\int_0^{t_{SL}} dt H_{tot}^{rf}} I_x \widehat{T} e^{i\int_0^{t_{SL}} dt H_{tot}^{rf}}\right\}, \quad \text{Eqn. (S4)}$$

since the initial and the measured operators (I_x) commute with U_{SL}.

The modified Eqn. (S1) is:

$$H_{tot}^{rf} = 2\pi \sum_{n=1}^{2} a_n \cos(n\omega_R t + \phi_n)\left(I_z \cos(\omega_{SL} t) + I_y \sin(\omega_{SL} t)\right)\widehat{Op}, \quad \text{Eqn. (S5)}$$

where $\widehat{Op}$ remains unchanged as it commutes with U_{SL}. The Eqn. (S5) can be rewritten in the following way:

$$H_{tot}^{rf} = \quad \text{Eqn. (S6)}$$

$$\pi \sum_{n=1}^{2} a_n \left[\left(\cos\left((n\omega_R + \omega_{SL})t + \phi_n\right) + \cos\left((n\omega_R - \omega_{SL})t + \phi_n\right)\right) I_z + \left(\sin\left((n\omega_R + \omega_{SL})t + \phi_n\right) - \sin\left((n\omega_R - \omega_{SL})t + \phi_n\right)\right) I_y\right] \widehat{Op}.$$

We see in Eqn. (S6) both $I_z\widehat{Op}$ and $I_y\widehat{Op}$ operators, which do not commute with the initial and final operators and are cosine or sine modulated. For small an, these terms can be approximated as zero, except for specific values of the spin lock frequency.

Under specific cases, when $k\omega_R - \omega_{SL} = 0$ (k=1 or 2), Eqn. (S6) can be rewritten as:

$$H^{rf}_{tot} = \pi a_k\big[\cos(\phi_n)I_z - \sin(\phi_n)I_y\big]\widehat{Op} + H^{rf}_{else}, \qquad \text{Eqn. (S7)}$$

while H^{rf}_{else} is:

$$H^{rf}_{else} = \pi\sum_{n=1}^{2} a_n\left[\cos\big((n+k)\omega_R t + \phi_n\big)I_z + \sin\big((n+k)\omega_R t + \phi_n\big)I_y\right]\widehat{Op}$$
$$+a_j\left[\cos\big((-1)^{k+1}\omega_R t + \phi_n\big)I_z - \sin\big((-1)^{k+1}\omega_R t + \phi_n\big)I_y\right]\widehat{Op}, \qquad \text{Eqn. (S8)}$$

where for the k=1 condition, j=2; and for the k=2 condition j=1. Eqn. (S7) can be further simplified using average Hamiltonian theory,(Haeberlen and Waugh, 1968) considering only the first-order term:

$$T_R {H^{rf}_{tot}}^{(0)} = \pi T_R a_k\big[\cos(\phi_n)I_z - \sin(\phi_n)I_y\big]\widehat{Op} = e^{-i\phi_n I_x}\frac{\pi a_k}{\nu_R} I_z\widehat{Op}e^{i\phi_n I_x}, \qquad \text{Eqn. (S9)}$$

where the average, ${H^{rf}_{tot}}^{(0)}$, is taken over one rotor period ($T_R = \frac{1}{\nu_R} = \frac{2\pi}{\omega_R}$). Regardless of the explicit form of the $\widehat{Op}$ operator, the measured spin-lock signal, according to Eqns. (S4) and (S9) is as follows:

$$S_{SL}(t_{SL} = N_{SL}T_R) \approx \cos\left(\pi\frac{a_k}{\nu_R}N_{SL}\right), \qquad \text{Eqn. (S10)}$$

since $e^{-i\phi_n I_x}$ commutes with the initial and final operators. For dipolar interactions, Eqn. (S10) should be modified to account for all orientations:

$$S_{SL}(t_{SL} = N_{SL}T_R) \approx \int d\Omega\cos\left(\pi\frac{a_k}{\nu_R}N_{SL}\right), \qquad \text{Eqn. (S11)}$$

The integration over orientation (Ω) indicates the powder averaging with Euler angles, (α, β, γ).(Mehring, 1983)

Figure S3 compares numerical (solid lines) and FOH curves (stars) under the rotary-resonance conditions $\nu_{SL} = \nu_R$ (Figures S3A-C) and $\nu_{SL} = 2\nu_R$ (Figures S3D-F) and different a_k

values, related either to B_0 modulation (Figures S1A and D), dipolar interaction (two-spin system, Figure S3B and E) or B_1 modulation (Figures S3C and F). Numerical simulations were performed using in-house MATLAB scripts based on the numerical solution of the equation of motion.(Nimerovsky and Goldbourt, 2012). These figures show full agreement between numerical and FOH curves. In all three cases, the changes in $a_k$values affect the modulation frequency of the spin-lock signal.

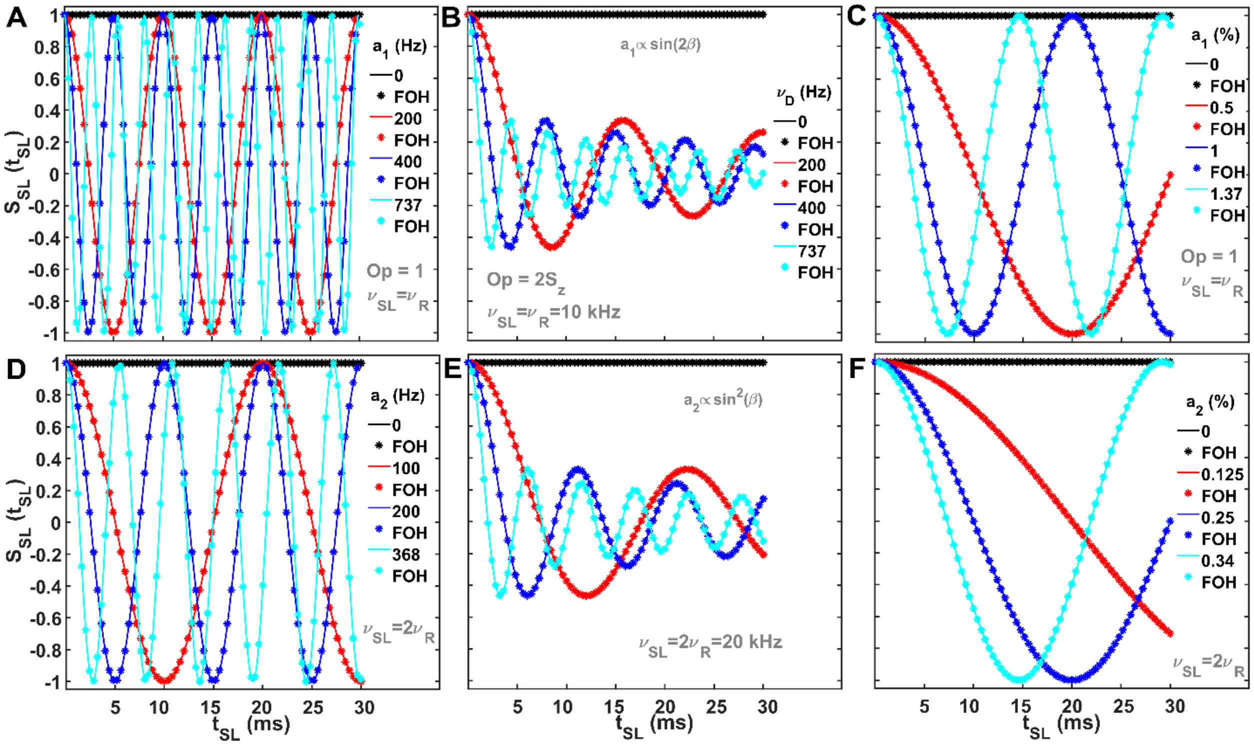

Figure S3 Numerical spin-lock (solid) and FOH (stars, Eqns. (S10) and (S11)) signals were simulated with different values of B_0 modulation (a single spin, A and D), dipolar coupling values (two spin-system, B and E) and B_1 modulation (a single spin, C and F) at rotary-resonance conditions, where $\nu_{SL} = \nu_R$ (A-C) and $\nu_{SL} = 2\nu_R$ (D-F). In (A) and (D), $a_2 = 0.5a_1$ with a_1: 0 – black; 200 Hz – red; 400 Hz – blue and 737 Hz – cyan. In (B) and (D), $a_1 = \frac{\nu_D}{\sqrt{2}}\sin(2\beta)$ and $a_2 = -\frac{\nu_D}{2}\sin^2(\beta)$ with dipolar coupling values ν_D of: 0 – black; 200 Hz – red; 400 Hz – blue and 737 Hz – cyan. In (C) and (F), $a_2 = 0.25a_1$ with a_1: 0 – black; 0.5% – red; 1% – blue and 1.37% – cyan. All simulations performed at 10 kHz MAS.

Dependence of the numerical (solid lines) and FOH curves (stars) on MAS rate is shown in Figure S4 under the conditions $a_1 = 2a_2 = 0.2$ kHz of B_0 modulation (Figures S4 A and D), $\nu_D = 0.2$ kHz of dipolar interaction (Figures S4 B and E) and $a_1 = 4a_2 = 1$ % of nominal B_1 (Figures S4 C and F), for both rotary-resonance conditions ($\nu_{SL} = \nu_R$ and $\nu_{SL} = 2\nu_R$). Only for B_1 modulation (Figures S4 C and F) does the change of MAS rate affect the modulation frequency. This is a simple consequence of B_1 modulation amplitude scaling up with B_1.

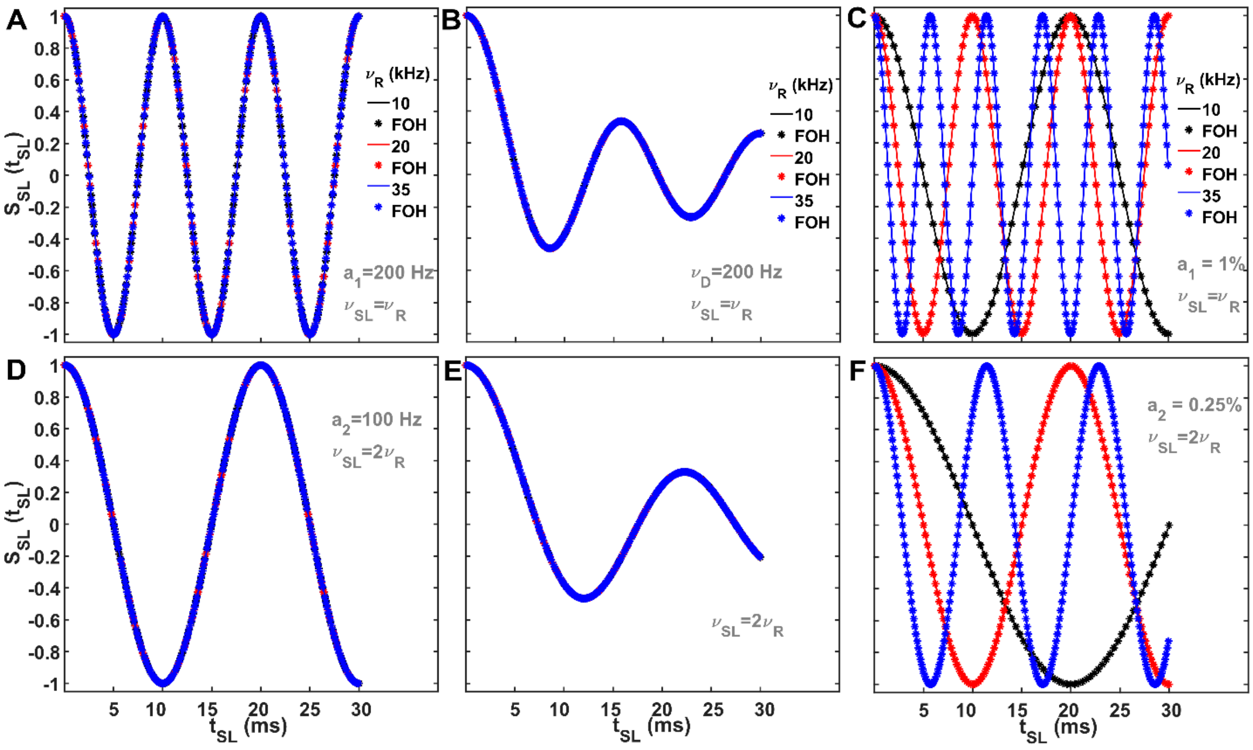

Figure S4 Numerical spin-lock (solid) and FOH (stars, Eqns. (S10) and (S11)) signals were simulated for B_0 modulation (a single spin, A and D), dipolar coupling values (two spin-system, B and E) and B_1 modulation (a single spin, C and F) at rotary-resonance conditions, where $\nu_{SL} = \nu_R$ (A-C) and $\nu_{SL} = 2\nu_R$ (D-F) under different ν_R: 10 kHz – black; 20 kHz – red; and 35 kHz – blue.

While Figure S4 shows spin-lock signals only at rotary-resonance conditions, Figure S5 presents numerical SL profiles for spin-lock strengths between 1 and 44 kHz under three different MAS rates: 10 kHz (A-C), 20 kHz (D-F) and 35 kHz (G-I) for B_0 modulation (A, D and

G), dipolar interaction (B, E and H) and B_0 modulation (C, E and I). The same conclusions as in Figure S4 are observed: for B_0 modulation (A, D and G) and dipolar interaction (B, E and H), the changes in MAS do not affect the modulation frequency, while for B_1 modulation (C, F and I), this is not the case (marked in gray in Figure S5). Additionally, the profiles show that the rotary-resonance conditions are narrow: a deviation of only 100 Hz from these conditions almost completely removes the influence of the time-dependent term on the spin-lock signal in all figures.

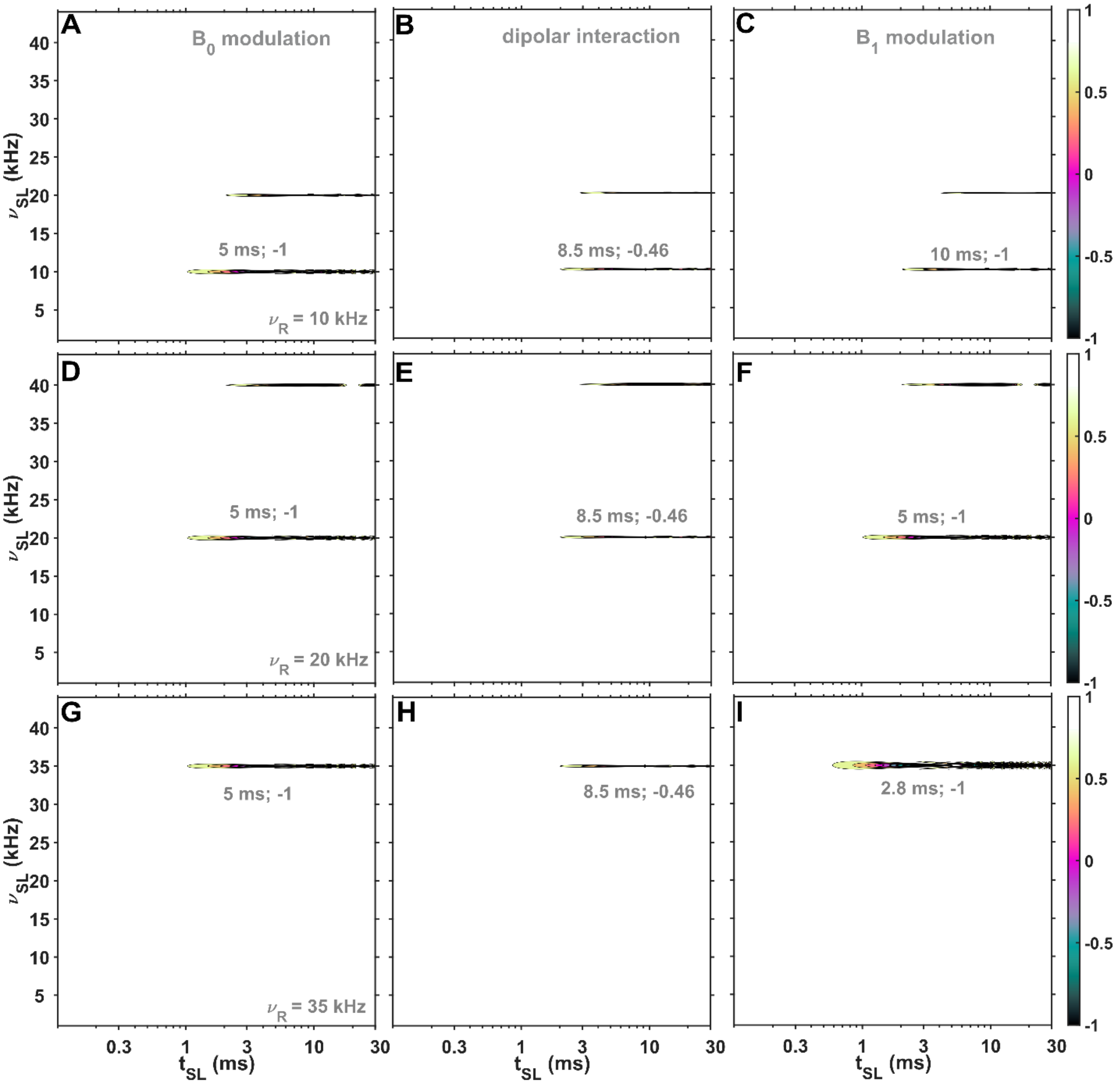

Figure S5 Numerical SL profiles showing the influence of time dependence introduced via B_0 modulation ($a_1 = 0.5a_2 = 100$ Hz, A, D and G), dipolar interaction ($\nu_D = 200$ Hz, B, E and H) and B_1 modulation ($a_1 = 0.25a_2 = 0.01\nu_{SL}$, C, F and I). The simulated signal is shown as a function of the rf-field strength (ν_{SL}, axis y) and mixing time (t_{SL}, axis x) of the SL under three different MAS rates: 10 kHz (A-C), 20 kHz (D-F) and 35 kHz (G-I). The values in gray represent the coordinates of the first minimum in the profiles. No phenomenological relaxation was included in the simulations.

In addition to time dependence induced by B_1 inhomogeneity, there is also time independent inhomogeneity,(Engelke, 2002; Gupta et al., 2015; Hoult, 1976; Paulson et al., 2004; Tošner et al., 2017, 2018) which is most clearly seen along the axis of the rotor (the spatial distribution of the applied ν_{SL} values to H_{SL}). Including this in the simulation broadens the rotary-resonance conditions. With addition of a time-independent term, parallel to the applied spin lock Hamiltonian becomes:

$$H_{SL} = 2\pi\left(\nu_{SL}I_x - \Delta_{B_1}\nu_{SL}I_x\right) = 2\pi\nu_{SL}\left(1-\Delta_{B_1}\right)I_x, \qquad \text{Eqn. (S12)}$$

where Δ_{B_1} represent the inhomogeneity factor for a given position in the sample. Experimentally, the reason of the appearance of this term is due to the fact that a solenoidal coil produces a higher rf-field at the center, as compared with the edges. Rather than explicitly averaging over the sample volume, we consider an approximate linear distribution of spin-lock signal. The average signal is then the sum of M signals with $\nu_{SL,l} = \nu_{SL}\left(1-(l-1)\frac{\Delta_{B_1}}{M}\right)$ $(l = 1, \ldots, M)$ and normalized with a Gaussian weighting factor,(Engelke, 2002; Gupta et al., 2015; Paulson et al., 2004; Xue et al., 2022) $g_{B_1,l}$:

$$S_{SL}(t_{SL}) = N_g \sum_{l=1}^{M} g_{B_1,l}\, \mathrm{Tr}\left\{I_x \hat{T} e^{-i\int_0^{t_{SL}} dt H_{total,l}} I_x \hat{T} e^{i\int_0^{t_{SL}} dt H_{total,l}}\right\}, \qquad \text{Eqn. (S13)}$$

where $\hat{T}$ is a Dyson operator, $g_{B_1,l} = e^{-\left(1.89\frac{(l-1)}{M}\right)^2}$ and N_g is a normalization factor.

The total Hamiltonian, $H_{total,l}$, is defined as follows:

$$H_{total,l} = \omega_{SL}\left(1-(l-1)\frac{\Delta_{B_1}}{M}\right)I_x + 2\pi\sum_{n=1}^{2} a_n \cos(n\omega_R t + \phi_n) I_z \widehat{Op}. \qquad \text{Eqn. (S14)}$$

In the following simulations, Δ_{B_1}=0.05 (5% with respect to applied ν_{SL}). While the value of Δ_{B_1} as well as the weighting factor can be different from coil to coil, this term is not the source of the appearance rotary-resonance conditions. However, this term broadens the rotary-resonance

conditions and alters the positions and values of the first minimum signal intensities, as shown in Figure S6.

As in the previous simulations, for B_0 modulation (A, D and G) and dipolar interaction (B, E and H), changes in MAS do not affect the modulation frequency, while for B_1 modulation (C, F and I), this is not the case. Additionally, for both B_0 modulation and dipolar interaction, the intensities at the first minima show a dependence on MAS rate, while for B_1 modulation, they do not (marked in gray in Figure S6). This differing dependence on MAS rate for B_1 modulation versus B_0 modulation and dipolar interaction could indicate which effect plays a major role in rotary-resonance condition experiments for isotropic samples. Since the rotary-resonance effect is observed for ^1H spins in 99.96% D_2O (Figure 3A-B in the main text), the dipolar interaction was not included in simulations in the main text.

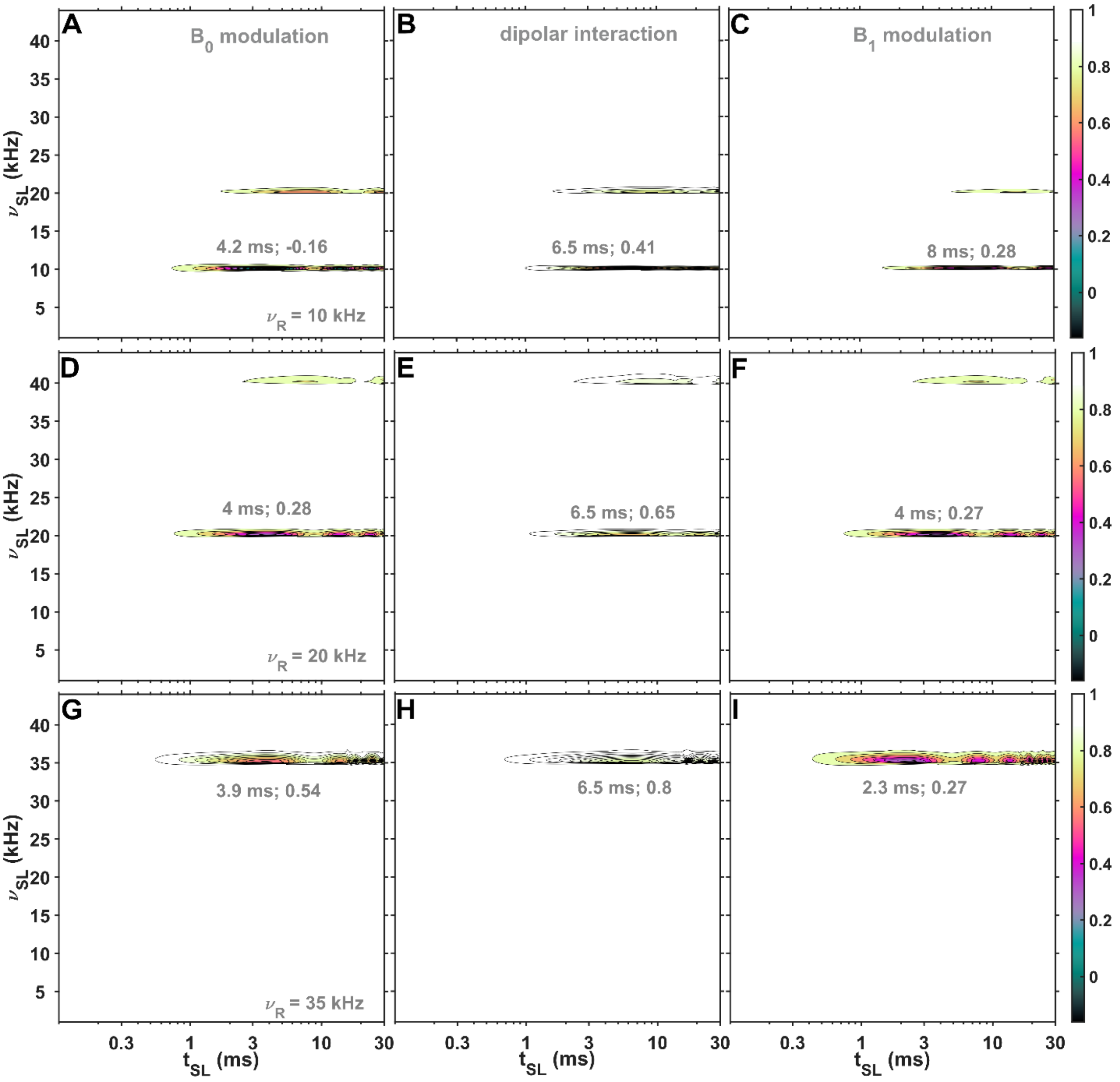

Figure S6 Numerical SL profiles with an additional time-independent term ($\Delta_{B_1} = 0.05$, Eqns. S12-S14) showing the influence of time dependence introduced via B_0 modulation ($a_1 = 0.5a_2 = 100$ Hz, A, D and G), dipolar interaction ($\nu_D = 200$ Hz, B, E and H) and B_1 modulation ($a_1 = 0.25a_2 = 0.01\nu_{SL}$, C, F and I). The simulated signal is shown as a function of the rf-field strength (ν_{SL}, axis y) and mixing time (t_{SL}, axis x) of the SL under three different MAS rates: 10 kHz (A-C), 20 kHz (D-F) and 35 kHz (G-I). The values in gray represent the coordinates of the first minimum in the profiles. No phenomenological relaxation was included in the simulations.

Time-dependent modulation may also be distributed. In that case, spin-lock signal will depend on the additional loop:

$$S_{SL}(t_{SL}) = N_{f,g}\sum_{k=1}^{W_x} f_{x,k}\sum_{l=1}^{M} g_{B_1,l}\,\mathrm{Tr}\left\{I_x\hat{T}e^{-i\int_0^{t_{SL}}dtH_{total,(k,l)}}I_x\hat{T}e^{i\int_0^{t_{SL}}dtH_{total,(k,l)}}\right\}, \qquad \text{Eqn. (S15)}$$

where $N_{f,g}$ is a normalization factor, x=B_1 or B_0. The total Hamiltonian, $H_{total,(k,l)}$, is defined as follows:

$$H_{total,(k,l)} = \omega_{SL}(1-G_{SL,l})I_x + \left\{2\pi A_{x,k}\sum_{n=1}^{2} a_n\cos(n\omega_R t+\phi_n)\right\}I_z\widehat{Op}, \qquad \text{Eqn. (S16)}$$

where x=B_1 or B_0.

Table S1 summarizes the amplitudes and the weighting factors for all time-dependent (B_0 and B_1 modulations) and time-independent (B_1) terms:

	a_n (in kHz)	The Amplitude	The weighting factor
B_0 modulation k=[1:26]	$a_1 = 2a_2 = 0.2$	$A_{B_0,k} = (k-1)/25$	$f_{B_0,k} = (k-1)/25$
B_1 modulation, time-independent l=[1:29]	-	$G_{SL,l} = (l-1)\frac{0.05}{28}$	$g_{B_1,l} = e^{-\left(1.89\frac{(l-1)}{28}\right)^2}$
B_1 modulation, time-dependent k=[1:29]	$a_1 = 4a_2 = 0.1\nu_{SL}$	$A_{B_1,k} = \left(1-e^{-\left(1.26\frac{(k-1)}{28}\right)^4}\right)$	$f_{B_1,k} = e^{-\left(1.26\frac{(k-1)}{28}\right)^4}$

Table S1 The summary of the amplitudes and the weighting factors for B_0 and B_1 modulations (the simulations are shown in Figures 4 and 5 in the main text).

For B_1 modulation, the bi-quadrate exponential distribution function does not represent the real distribution of the time-dependent B_1 modulation; it simply provides non-linear sampling, different from the time-independent term. Figure S7 depict the weighting factors as functions of amplitudes

for time-dependent B_0 (Figure S7A), time-dependent B_1 (Figure S7B) and time-independent B_1 (Figure S7C).

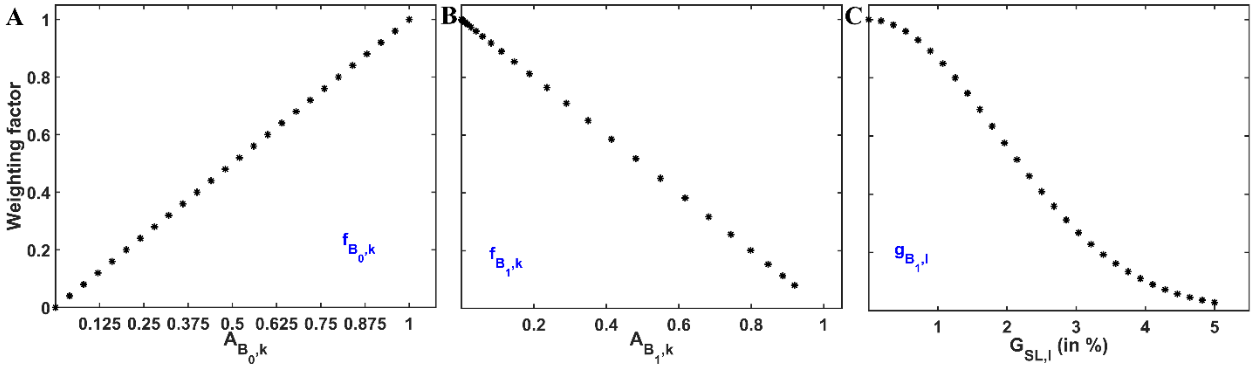

Figure S7 The weighting factors (axis y) and the amplitudes (axis x) for simulations of time-dependent B_0 ($f_{B_0,k}$, A) and B_1 ($f_{B_1,k}$, B). (C) displays the weighting factor for time-independent rf-field inhomogeneity ($G_{SL,l}$).

Figure S8 shows the weighting factors (the color map) according to the range of input inhomogeneity factor values (y-axis) and B_0 modulation amplitude values (x-axis, A) and B_1 modulation amplitude values (x-axis, B). For B_0 modulation (A), the amplitude with the highest value of 200 Hz has the largest weighting factor (red), while for B_1 modulation (B), the opposite is true – the amplitude with 0 value has the largest weighting factor on the total signal. This approximates the expectation that a B_0 field inhomogeneity (e.g. in the y direction) would more strongly affect the sample at the rotor wall, where there is more sample volume. On the other hand, the B_1 field inhomogeneity may be less linear, and affect only a small annulus of sample near the coil. Changes to the distribution mainly affect the location of the first minimum in the SL intensity (see Figure S6 and Figures 4 and 5 in the main text).

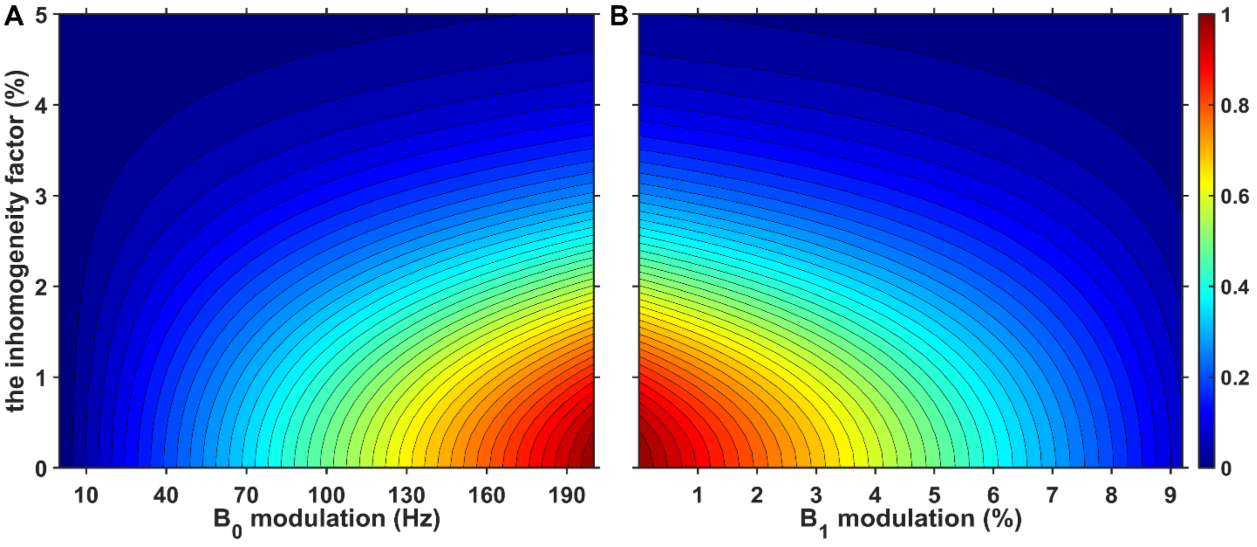

Figure S8 The weighting factor maps are plotted as a function of the inhomogeneity factor (y-axis, in %) and B_0 (A) or B_1 (B) amplitude modulations (x-axis). (A) The amplitudes values range between [0:200] (Hz). (B) The amplitude values range between [0:9.14] (%) with respect to the applied ν_{SL} value. In both color maps, the inhomogeneity factor ranges between [0:5] (%) with respect to the applied ν_{SL} value.

In the main text, Figures 4 and 5 show the simulated SL-lock profiles where time-dependent (B_1 or B_0 modulations) and time-independent (the inhomogeneity) are simulated with the distribution (Table S1).

Sample preparation

Commercial polybutadiene rubber with ^{13}C at natural abundance was cut in a single piece to fill a 1.3 mm MAS rotor. For the commercial 99.96% D_2O, 5 mM of Cu^{2+} ethylenediaminetetraacetic acid disodium salt was added to accelerate the acquisition. The liquid water was then placed into a 1.3 mm MAS rotor.

A 4 mm MAS rotor was filled with 20 percent polyethylene glycol solution in water with ^{13}C at natural abundance. Specifically, 26 mg of PEG was dissolved in 250 µL D_2O (10 %). This

sample was filled in a 4 mm Bruker zirconium oxide (ZrO2) rotor and the cap was glued on top with two component epoxy to prevent leakage during spinning

Solid NMR Spectroscopy

1.3 mm probe: Spin-Lock experiments were conducted on a Bruker Avance III HD spectrometer operating at 14.1 T (600 MHz ^{1}H frequency) using a DVT600W2 BL1.3 mm HXY probe. The experiments were performed at static, 10, 20 and 35 kHz MAS. The temperature of the nitrogen cooling gas set to 289 K, with 300 liters per hour.

D_2O. The experiments were performed without decoupling. In each experiment, 8 scans were recorded. The delay time between single experiments was set to 2.8 s. For determination of the reference rf-field ^{1}H power, the 1D ^{1}H signal was recorded as a function of the length of a single pulse (on resonance).

Polybutadiene rubber. For decoupling of the heteronuclear j-coupling, WALTZ-16 was used:(Shaka et al., 1983) 80 μs 2-π pulses (12 kHz rf-field strength). In each experiment, 56 scans were recorded. The delay time between single experiments was set to 2 s. For determination of the reference rf-field ^{13}C power, the 1D ^{13}C signal was recorded as a function of the length of a single pulse (on resonance).

4mm probe: Spin-Lock experiments were conducted on a Bruker Avance III HD spectrometer operating at 14.1 T (600 MHz ^{1}H frequency) using a 4 mm HXY probe. The experiments were performed at 10 kHz MAS. The temperature of the nitrogen cooling gas was set to 290 K, with 300 liters per hour.

Polyethylene glycol solution. SW_f-TPPM was used for decoupling of the heteronuclear j-coupling:(Thakur et al., 2006) 120 μs for single ~π-pulse and 6.3 kHz rf-field strength. In each

experiment, 64 scans were recorded. The delay time between single experiments was set to 2 s. For determination of the reference rf-field ^{13}C power, 1D ^{13}C signal was recorded as a function of the length of a single pulse.

REFERENCES

Aebischer, K., Tošner, Z., and Ernst, M.: Effects of radial radio-frequency field inhomogeneity on MAS solid-state NMR experiments, Magn. Reson., 2, 523–543, https://doi.org/10.5194/mr-2-523-2021, 2021.

Engelke, F.: Electromagnetic wave compression and radio frequency homogeneity in NMR solenoidal coils: Computational approach, Concepts Magn. Reson., 15, 129–155, https://doi.org/10.1002/cmr.10029, 2002.

Gupta, R., Hou, G., Polenova, T., and Vega, A. J.: RF Inhomogeneity and how it Control CPMAS, Solid State Nucl. Magn. Reson., 72, 17–26, https://doi.org/10.1016/j.ssnmr.2015.09.005, 2015.

Haeberlen, U. and Waugh, J. S.: Coherent Averaging Effects in Magnetic Resonance, Phys. Rev., 175, 453–467, https://doi.org/10.1103/PhysRev.175.453, 1968.

Hahn, E. L.: Spin Echoes, Phys. Rev., 80, 580–594, https://doi.org/10.1103/PhysRev.80.580, 1950.

Hoult, D. I.: Solvent peak saturation with single phase and quadrature fourier transformation, J. Magn. Reson. 1969, 21, 337–347, https://doi.org/10.1016/0022-2364(76)90081-0, 1976.

Mehring, M.: Principles of High Resolution NMR in Solids, 2nd ed., Springer-Verlag, Berlin Heidelberg, https://doi.org/10.1007/978-3-642-68756-3, 1983.

Nimerovsky, E. and Goldbourt, A.: Insights into the spin dynamics of a large anisotropy spin subjected to long-pulse irradiation under a modified REDOR experiment, J. Magn. Reson., 225, 130–141, https://doi.org/10.1016/j.jmr.2012.09.015, 2012.

Olejniczak, E. T., Vega, S., and Griffin, R. G.: Multiple pulse NMR in rotating solids, J. Chem. Phys., 81, 4804–4817, https://doi.org/10.1063/1.447506, 1984.

Paulson, E. K., Martin, R. W., and Zilm, K. W.: Cross polarization, radio frequency field homogeneity, and circuit balancing in high field solid state NMR probes, J. Magn. Reson., 171, 314–323, https://doi.org/10.1016/j.jmr.2004.09.009, 2004.

Shaka, A. J., Keeler, J., Frenkiel, T., and Freeman, R.: An improved sequence for broadband decoupling: WALTZ-16, J. Magn. Reson. 1969, 52, 335–338, https://doi.org/10.1016/0022-2364(83)90207-X, 1983.

Thakur, R. S., Kurur, N. D., and Madhu, P. K.: Swept-frequency two-pulse phase modulation for heteronuclear dipolar decoupling in solid-state NMR, Chem. Phys. Lett., 426, 459–463, https://doi.org/10.1016/j.cplett.2006.06.007, 2006.

Tošner, Z., Purea, A., Struppe, J. O., Wegner, S., Engelke, F., Glaser, S. J., and Reif, B.: Radiofrequency fields in MAS solid state NMR probes, J. Magn. Reson., 284, 20–32, https://doi.org/10.1016/j.jmr.2017.09.002, 2017.

Tošner, Z., Sarkar, R., Becker-Baldus, J., Glaubitz, C., Wegner, S., Engelke, F., Glaser, S. J., and Reif, B.: Overcoming Volume Selectivity of Dipolar Recoupling in Biological Solid-State NMR Spectroscopy, Angew. Chem. Int. Ed., 57, 14514–14518, https://doi.org/10.1002/anie.201805002, 2018.

Vold, R. L., Waugh, J. S., Klein, M. P., and Phelps, D. E.: Measurement of Spin Relaxation in Complex Systems, J. Chem. Phys., 48, 3831–3832, https://doi.org/10.1063/1.1669699, 1968.

Xue, K., Nimerovsky, E., Tekwani Movellan, K. A., Becker, S., and Andreas, L. B.: Backbone Torsion Angle Determination Using Proton Detected Magic-Angle Spinning Nuclear Magnetic Resonance, J. Phys. Chem. Lett., 13, 18–24, https://doi.org/10.1021/acs.jpclett.1c03267, 2022.
