# Supplementary material for: Pseudo rotary resonance relaxation dispersion effects in isotropic samples
Source: Magn Reson (Gott). 2025 Jun 3;6(1):119–29. doi: 10.5194/mr-6-119-2025 (PMC12247080; doi:10.5194/mr-6-119-2025)
Supplement: The supplement related to this article is available online at https://doi.org/10.5194/mr-6-119-2025-supplement. [file mr-6-119-2025-supplement.zip › mr-6-119-2025-supplement-title-page.pdf]

Supplement of Magn. Reson., 6, 119–129, 2025
https://doi.org/10.5194/mr-6-119-2025-supplement
© Author(s) 2025. CC BY 4.0 License.

Supplement of

Pseudo rotary resonance relaxation dispersion effects in isotropic samples

Evgeny Nimerovsky et al.

Correspondence to: Evgeny Nimerovsky (evni@mpinat.mpg.de) and Loren B. Andreas (land@mpinat.mpg.de)

- mr-6-119-2025-supplement-title-page.pdf
- MALTLAB_codes
  - 100.dat
  - ID.m
  - pseudo_RRD_comparison_with_FOH.m
  - pseudo_RRD_spin_lock_profiles_B0_B1_modulations.m
- pseudoRRD_revII_SI.pdf

The copyright of individual parts of the supplement might differ from the article licence.
